# Mechanisms of nitrogen transfer in a model clover-ryegrass pasture: a ^15^N-tracer approach

**DOI:** 10.1007/s11104-022-05585-0

**Published:** 2022-07-28

**Authors:** Michaela K. Reay, Katrina A. Pears, Alison Kuhl, Richard P. Evershed, Phillip J. Murray, Laura M. Cardenas, Jennifer A. J. Dungait, Ian D. Bull

**Affiliations:** 1 Organic Geochemistry Unit, School of Chemistry, University of Bristol, Cantock’s Close, Bristol, BS8 1TS UK; 2 Department of Sustainable Agriculture Sciences, Rothamsted Research - North Wyke, Okehampton, EX20 2SB Devon UK; 3 Present Address: School of Agriculture, Food and Environment, Royal Agricultural University, Cirencester, GL7 6JS UK; 4 Present Address: Carbon Management Center, SRUC - Scotland’s Rural College, Edinburgh, Scotland EH9 3JG UK; 5 Present Address: Geography, CLES - Amory Building, University of Exeter, Exeter, EX4 4RJ UK

**Keywords:** Nitrogen transfer, Clover, ^15^N-stable isotope probing, Soil microbial community

## Abstract

**Purpose:**

Nitrogen (N) transfer from white clover (*Trifolium repens* cv.) to ryegrass (*Lolium perenne* cv.) has the potential to meet ryegrass N requirements. This study aimed to quantify N transfer in a mixed pasture and investigate the influence of the microbial community and land management on N transfer.

**Methods:**

Split root ^15^N-labelling of clover quantified N transfer to ryegrass via exudation, microbial assimilation, decomposition, defoliation and soil biota. Incorporation into the microbial protein pool was determined using compound-specific ^15^N-stable isotope probing approaches.

**Results:**

N transfer to ryegrass and soil microbial protein in the model system was relatively small, with one-third arising from root exudation. N transfer to ryegrass increased with no microbial competition but soil microbes also increased N transfer via shoot decomposition. Addition of mycorrhizal fungi did not alter N transfer, due to the source-sink nature of this pathway, whilst weevil grazing on roots decreased microbial N transfer. N transfer was bidirectional, and comparable on a short-term scale.

**Conclusions:**

N transfer was low in a model young pasture established from soil from a permanent grassland with long-term N fertilisation. Root exudation and decomposition were major N transfer pathways. N transfer was influenced by soil biota (weevils, mycorrhizae) and land management (e.g. grazing). Previous land management and the role of the microbial community in N transfer must be considered when determining the potential for N transfer to ryegrass.

**Supplementary Information:**

The online version contains supplementary material available at 10.1007/s11104-022-05585-0.

## Introduction

The productivity of agricultural systems must be improved to satisfy the increased demand from the growing global population, with 70 to 100% more food required by 2050 (Tilman et al. 2011; Ray et al. 2013). This heightened demand is frequently met by increasing nitrogen (N) fertiliser applications, with N often the nutrient which limits crop growth. However, it is unsustainable and environmentally damaging to continually over-apply N fertiliser to increase productivity (Vitousek et al. 2009). N pollution from agriculture contributes to eutrophication of terrestrial and aquatic systems, acidification of soils, global warming and ozone depletion (Anderson et al. 2002; Fowler et al. 2013). Furthermore, the high profile criticism of the carbon footprint of livestock production is driving an imperative to reduce fertiliser inputs in intensively managed systems (Herrero et al. 2016). An average of 355 thousand tonnes of N fertiliser are applied to UK grasslands every year, averaging 54 kg N ha^−1^ yr^−1^ (DEFRA 2020). Therefore, there is renewed interest in the use of N fixing legumes to improve N supply to non-legumes (Paynel et al. 2008; Fustec et al. 2010; Thilakarathna et al. 2016).

The ability of legumes to form symbiotic relationships with rhizobia makes them a key component of mixed swards. Clover (*Trifolium repens* L.) is capable of fixing N equivalent of up to 150 kg N ha^−1^ y^−1^ (Elgersma and Hassink 1997). The fixed N is subsequently bioavailable and can be transferred via a range of pathways to non-legumes, contributing between 0 to 80% of non-legume N (Høgh-Jensen and Schjoerring 1997; Moyer-Henry et al. 2006; Rasmussen et al. 2007; Gylfadóttir et al. 2007; Chalk et al. 2014). The importance of plant N transfer, and the contribution of individual N transfer pathways, is dependent on the influence of rhizodeposits on mineralisation-immobilisation-turnover (MIT) in soil, competition between plants and the soil microbial community, the capacity for N uptake by the receiving plant, and availability of other N sources (Jensen 1996). Optimisation of N transfer has the potential to increase crop yields via improved soil fertility and nutrient status, providing high quality feed for grazing animals, and maximise the benefits of mixed intercropping systems (Haynes 1980; Thilakarathna et al. 2016), as well as reducing the environmental effects of reactive N (Galloway et al. 2013). Utilising legume N fixation as a source of N for forage crops, e.g., ryegrass (*Lolium perenne* L.) in sustainable grassland management is currently limited by poor understanding of the relative importance of different N transfer pathways within a mixed sward (Thilakarathna et al. 2016). To maximise the benefits of the association between legumes and non-legumes, a better understanding of the underlying mechanisms and factors which govern N transfer is needed. There are a number of biotic N transfer pathways between legumes and non-legumes, which are also influenced by management practices. Fixed nitrogen, e.g., as ammonium and amino acids has been shown to significantly contribute to total N rhizodeposition in early-growth stages of legumes (Lesuffleur et al. 2013). Root exudates are either directly incorporated into non-legumes, with uptake of inorganic and organic N (Näsholm et al. 2009), or via MIT by soil microbes, facilitating indirect N transfer of root exudates (van Kessel et al. 2009; Jalonen et al. 2009). Furthermore, root exudates contain other compounds which stimulate arbuscular mycorrhizal fungi (AMF) symbiosis (e.g., flavonoids, strigolactones), another key pathway of N transfer (Steinkellner et al. 2007; Wahbi et al. 2016; Thilakarathna et al. 2016; Coskun et al. 2017). Similar to root exudates, mycorrhizae influence N transfer directly, via mycelial connections between legumes and non-legumes (Høgh-Jensen 2006; Meng et al. 2015), and indirectly, by N uptake into the mycorrhizal system, reducing N losses. Retained N can subsequently be transferred to non-legumes (Hodge and Fitter 2010; Asghari and Cavagnaro 2012). AMF, alongside the soil microbial community, support decomposition of senescing legume tissues providing N for non-legumes (Fustec et al. 2010). This is indirect, requiring MIT prior to uptake by non-legumes, but it has been estimated 2 to 26% of biologically fixed N can be transferred via decomposition of roots and nodules (Ledgard and Steele 1992; Jørgensen et al. 1999; Wichern et al. 2008; Louarn et al. 2015). Given the slower nature of this pathway, it contributes to N transfer in the latter stages of plant growth. This pathway incorporates decomposition of both above and belowground legume tissue, and is influenced by the management of mixes pastures. Grazing will remove aboveground biomass for decomposition, but the majority will be returned as excretion (Thilakarathna et al. 2016). Furthermore, defoliation has the potential to increase decomposition of remaining belowground legume tissues, promoting N transfer via this pathway (Ledgard 2001; Dahlin and Stenberg 2010; Peoples et al. 2015). The influence of plant pests on N transfer has also been investigated. For example, *Sitona* sp. weevils, which are common invertebrate pests of white clover, cause increased N transfer following clover root damage, reduced efficiency of N uptake and increased decomposition of sloughed roots and promote ‘leakage’ of N from legume roots (Murray and Clements 1998; Murray et al. 2002). Through these biotic pathways, with potential influences from land management, N transfer from non-legumes to legumes accounts for 0–8% of legume N, while non-legumes can have up to 80% of N derived from N transfer from legumes (Høgh-Jensen and Schjoerring 2000; Paynel and Cliquet 2003; Rasmussen et al. 2007; Gylfadóttir et al. 2007; Jamont et al. 2013).

The complexity of N transfer between legumes and non-legumes belowground means that it is challenging to directly determine the importance of individual N transfer pathways, particularly as they are inter-dependent. Reducing the complexity of the grassland N-cycle can help to elucidate the relative importance of different N transfer pathways, in particular by controlling abiotic factors (e.g. irrigation, temperature, N fertiliser application and soil N) to quantify biotic N transfer pathways (Chalk et al. 2014; Thilakarathna et al. 2016). A number of experimental approaches have been applied to quantify N transfer between legumes and non-legumes, as reviewed by Chalk et al. (2014). The two most common methods that are suitable for both field and laboratory experiments are ^15^N isotope dilution and ^15^N-shoot labelling. However, the dilution method is indirect, time-limited (due to uptake of applied ^15^N fertiliser by clover and subsequent exudation) and has high observed variability (Barraclough 1995; Viera-Vargas et al. 1995; Chalk et al. 2014). ^15^N-shoot labelling is direct, although it is not a natural N uptake pathway in grasslands and little attention has been paid to the potential alteration of root dynamics and subsequent N transfer (Chalk et al. 2014). ^15^N-labelling via natural pathways of N_2_ fixation using ^15^N_2_ (e.g. (Frey and Schuepp 1992) and root uptake, via transplantation (e.g. (Carlsson and Huss-Danell 2014) or split root labelling (e.g. Martin et al. 1991; Johansen and Jensen 1996), enables continuous ^15^N labelling. While not suitable for a field setting, split root labelling has provided comparable N transfer estimates to shoot labelling (Martin et al. 1991) and can be used to quantify individual N transfer pathways under controlled conditions (Wichern et al. 2008; Chalk et al. 2014; Thilakarathna et al. 2016). One caveat must be the disturbance of roots during set-up of split-root labelling, which may influence N transfer pathways, particularly exudation, mycorrhizal networks and root-root contact, which must be considered during experimental design. However, this method is suitable for quantifying N transfer to the soil microbial community, an important competing fate for legume-N, which also acts as an N transfer pathway during microbial decomposition and turnover, and plays a key role in indirect N transfer of exudates, for mycorrhizal pathways and legume tissue decomposition (Ta and Faris 1987; Parton et al. 2007; Charteris et al. 2016). Within the soil N pool, proteinaceous N accounts for up to 60%, and plays a key role in microbial metabolic activity (e.g., enzymes, structures, transport Schulten and Schnitzer 1998; Friedel and Scheller 2002). Targeting this pool allows direct interrogation of the biomolecular fate of N following N fixation (e.g. Chiewattanakul et al. 2022), and during N transfer from legumes to non-legumes. Recent developments in novel compound-specific ^15^N-stable isotope probing (SIP) approaches have the potential to enable the direct quantification of clover-N transfer to the soil microbial protein pool (Charteris et al. 2016).

This study aimed to investigate near-to-natural ecosystem N transfer between clover and ryegrass using split-root ^15^N-labelling of clover. N transfer to ryegrass was quantified following manipulation of N transfer pathways including the addition of soil biota proposed to promote N transfer (e.g. mycorrhizae, below ground herbivores), mimicking of agricultural practices (e.g. grazing and cover crop incorporation), and sterilisation of soil to remove competition for N from the soil microbial community. This approach allowed direct quantification for N transfer pathways (e.g. exudation). N transfer was determined using bulk ^15^N analyses and compound-specific ^15^N analyses of the soil protein pool, the latter providing essential information regarding the role of the soil microbial community in N transfer and competition with non-legumes for legume-derived N in a mixed cropping system. We hypothesise that (i) N transfer to ryegrass will be an important source of available N for ryegrass and soil microbes, via both direct and indirect transfer of clover-N, (ii) N transfer will be supported by the soil microbial community during decomposition and through mycorrhizae, (iii) damage to clover via pests or defoliation will increase N transfer. This has the potential for use in the development of more sustainable agricultural strategies to support crop N supply and reduce fertiliser N input requirements and associated environmental costs currently limited by the lack of understanding regarding N transfer pathways (Wahbi et al. 2016; Thilakarathna et al. 2016).

## Methods

### Soil collection

Soil was collected from a permanent grazed grassland system at the North Wyke Farm Platform (Okehampton, Devon, UK; 50°46’N, 3°54’W). The soil is a clay loam topsoil (36% clay) and the vegetation was *Lolium* spp. interspersed with *Cynosurus, Festuca, Agrostis, Holcus* and *Dactylis* spp. (Bol et al. 2004; Harrod and Hogan 2008; Peukert et al. 2012; Orr et al. 2016). The mean annual rainfall is 1055.7 mm with a mean annual temperature of 9.6 °C (Harrod and Hogan 2008). All farmlets were managed in the same way and received 200 kg N ha^−1^ of N fertiliser alongside P, K and S before cutting (Orr et al. 2016). Soil cores (264; 10 cm depth, 5.5 cm diameter) were collected on a 50 m grid (Figure S1) across the Farm Platform in June and July 2012. Soil was air-dried and sieved to 2 mm with vegetation and stones removed to give a composite sample and stored at 4 °C until required for use. Soil total carbon (TC) was 5.23% and total nitrogen (TN) was 0.57%. Soil pH was 6.3. Acid washed horticultural silver sand was sieved (1 mm) and furnaced at 450 °C.

### N transfer experiments

All plants used in the study were from white clover (*Trifolium repens* L.) and perennial ryegrass (*Lolium perenne* cv) mother plants established from seed in soil, and plants used in subsequent experiments were all from the same mother plants to reduce plant genetic variations. Experiments were conducted in a temperature-controlled greenhouse (20 °C) with a photoperiod of 16 h. Additional lighting was provided when natural lighting was below 30 W m^−2^ and shading was provided when natural light was above 220 W m^−2^. Plants were watered daily with tap water and weekly with a modified nutrient solution adapted from (Hewitt 1966); omitting N, one-fifth strength; Table S1) prior to the experimental period.

**Fig. 1:**
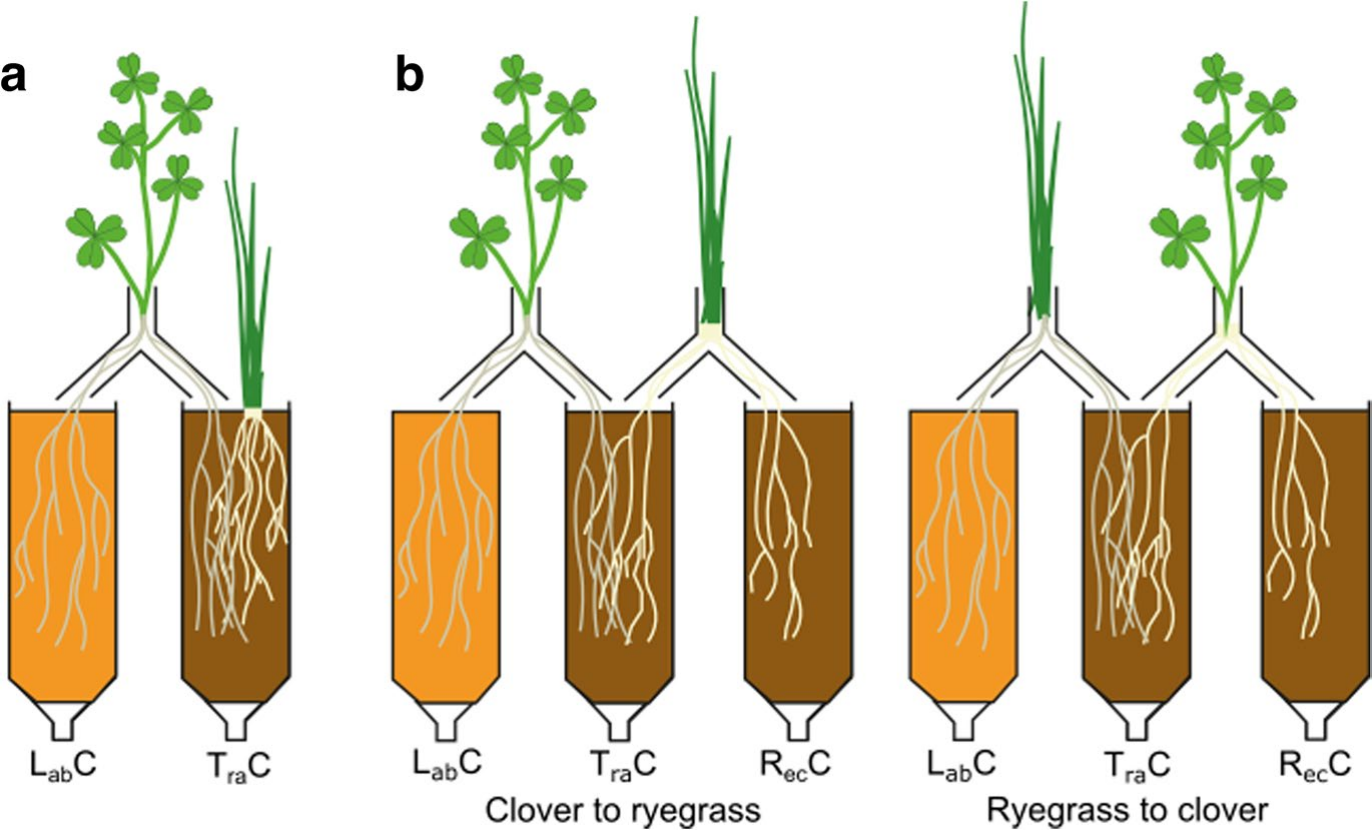
Experimental set-up of split root ^15^N-labelling technique. The labelling compartment (L_ab_C), where the ^15^N-urea was added, contained sand (orange) and transfer compartment (T_ra_C) and receiving compartment (R_ec_C) contained soil (brown). |Part (**a**) shows the two-compartment set-up used. Clover roots were split across the L_ab_C and T_ra_C and grass roots were all in the T_ra_C. Part (**b**) shows the three-compartment set-up used, with clover roots split evenly between the L_ab_C and T_ra_C, and ryegrass roots split evenly between T_ra_C and R_ec_C for the clover to ryegrass experiment. For the ryegrass to clover experiment, ryegrass roots were split between the L_ab_C and T_ra_C, and clover roots between T_ra_C and R_ec_C. Diagram is not to scale

A split root ^15^N-labelling technique was used to determine N transfer between plants which enabled ^15^N incorporation by a natural N uptake process and ensured all N compounds subject to transfer were ^15^N labelled (Wichern et al. 2008; Chalk et al. 2014). Established clover and grass plants were rooted into either sand (25 g) and soil (15 g) compartments (10 cm height, 2 cm diameter), with clover roots evenly split between the labelling and transfer compartments (L_ab_C and T_ra_C, respectively; Fig. 1) and allowed to establish for 3 weeks prior to treatment. ^15^N-urea was applied in five of the eight treatments to the L_ab_C, shown in Table 1. It was not applied in the negative control, or exudate and decomposition treatment, where ^15^N-labelled inputs (exudates and clover shoots, respectively) were used. The base of the compartments was tapered and plugged with furnaced glass wool to maintain aerobic conditions throughout the soil profile. During incubation, compartments were maintained at 60% water holding capacity (WHC) using full strength nutrient solution daily (Table S1; (Hewitt 1966). Losses (e.g. leaching or gaseous) were limited by the experimental design by constant soil moisture.

**Table 1:** Summary of compartments and treatments. n/a = this compartment was not used for this particular treatment. DDW = double distilled water; FWt = fresh weight; L_ab_C = labelling compartment, T_ra_C = transfer compartment, R_ec_C = receiving compartment

Treatment	L_ab_C	T_ra_C	Description of treatment
(i) Controls			
No ^15^N addition	DDW + Sand	Soil	1 ml of DDW
Sterile	^15^N-Urea + Sand	Soil	Autoclaved soil used in T_ra_C
(ii) Land management / N type			
^15^N-urea only	^15^N-Urea + Sand	Soil	^15^N-urea only (1 ml 30 mM 98 atom% ^15^N)
Decomposition	n/a	^15^N-labelled clover incorporated in soil	^15^N-labelled clover shoots (2.1 ± 0.3 atom% ^15^N) produced via labelling with ^15^N urea (1 ml 30 mM 98 atom% ^15^N) Finely chopped clover shoot (0.7 g FWt) incorporated into T_ra_C
Exudation	n/a	Soil watered with ^15^N-labelled root exudates	T_ra_C watered with leachate containing ^15^N-labelled clover root exudates produced following clover labelling with ^15^N-urea(1 ml 30 mM 98 atom% ^15^N)
Defoliation	^15^N-Urea + Sand	Soil	Clover shoots removed at 100 h
(iii) Presence of organisms			
Fungi	^15^N-Urea + Sand	Soil	^15^N-urea (1 ml 30 mM 98 atom% ^15^N) in L_ab_C Fungi (*Rhizophagus irregularis*) on a growth medium (1.5 g) was mixed into the T_ra_C
Weevil	^15^N-Urea + Sand	Soil	^15^N-urea (1 ml 30 mM 98 atom% ^15^N) in L_ab_C 20 Weevil eggs (*Sitona* spp.) were injected into the soil T_ra_C
(iv) Bidirectional N transfer			
Clover to ryegrass	^15^N-Urea + Sand	Soil	^15^N-urea (1 ml 30 mM 98 atom% ^15^N) in L_ab_C Receiver (ryegrass) split across T_ra_C and R_ec_C. R_ec_C contained soil
Ryegrass to clover	^15^N-Urea + Sand	Soil	^15^N-urea (1 ml 30 mM 98 atom% ^15^N) in L_ab_C Receiver (clover) split across T_ra_C and R_ec_C. R_ec_C contained soil

The one-and two-compartment experiments were classified into three categories: (i) controls; (ii) land management/ N type and (iii) presence of organisms, shown in Table 1. Each treatment had four replicates. Two control experiments were conducted. The first added no ^15^N-label, with double distilled water (DDW; 1 ml) added to the L_ab_C. The second control experiment used sterilised soil, via autoclaving, with ^15^N-urea (1 ml, 30 mM of 98 atom% ^15^N) applied to L_ab_C. For land management treatments / N type incubations, one treatment only applied ^15^N urea to L_ab_C (1 ml 30 mM of 98 atom% ^15^N). This treatment quantified N transfer in the experiment when all natural pathways were present, and there was no other controls on N transfer (e.g., grazing, pests), while also representing fertiliser addition to the pasture. The decomposition treatment mixed finely chopped clover shoots (2.1 ± 0.3 atom% ^15^N, 0.7 g FWt), which were collected from clover grown separately with ^15^N urea (1 ml 30 mM of 98 atom% ^15^N), into T_ra_C soil. The root exudate treatment applied root exudates collected from ^15^N-labelled clover into the T_ra_C soil. Root exudates were obtained from clover grown across two sand-filled compartments, with ^15^N-urea (1 ml 30 mM of 98 atom% ^15^N) applied to one, and root exudates collected from the second. The exudates were collected by leaching and immediately used to water the T_ra_C in the exudate treatment. Finally, the defoliation treatment mimicked grazing, by removal of clover shoots 100 h after application of ^15^N-urea (1 ml 30 mM of 98 atom% ^15^N). The third category, presence of organisms, added either *Sitona* spp. or AMF (*Rhizophagus irregularis*) into the T_ra_C with ^15^N-urea (1 ml 30 mM of 98 atom% ^15^N) added to the L_ab_C. This AMF was selected as it is commonly used in agriculture, can be found in most soils and has a wide range of host plants. All incubations were halted at 480 h when aboveground biomass was removed immediately to stop N transfer. Roots were removed from the compartments as an intact root system attached to the shoot, separated from above ground biomass, and washed with DDW to remove soil. Additionally, the soil was handpicked for any roots broken during this process. Shoots, roots and soil were frozen (−20 °C), freeze-dried and plant biomass determined.

N transfer from clover to ryegrass and vice versa was determined using an additional receiving compartment (R_ec_C; Fig. 1b). In both directions, the donor plant roots were evenly split between the L_ab_C and T_ra_C, and the receiver plant roots were split between the T_ra_C and R_ec_C. In both treatments (*n* = 4), ^15^N-urea (1 mL of 30 mM 98 atom % ^15^N urea) was applied to the L_ab_C and the incubation was halted at 100 h. Clover and ryegrass root exudates in the clover to ryegrass, and ryegrass to clover experiments, respectively, were collected from the L_ab_C by leaching with DDW (40 ml), to avoid contaminations from soil free amino acids (FAAs) in the T_ra_C. Leachate was frozen and freeze-dried for amino acid (AA) preparation and analysis. This procedure was repeated with sand (80 g) and DDW (80 mL) with a mixture of AA standards (100 µL of 1 mg mL^−1^ for each AA; Table S7) to determine recovery of AAs from sand.

### Extraction, isolation and derivatisation of hydrolysable AAs from soil

Extraction, isolation and derivatisation of soil AAs were conducted as described by Charteris et al. (2016). Freeze-dried soil (100 mg) was hydrolysed with 6 M HCl (5 mL; 100 °C for 24 h) under an N_2_ atmosphere to yield total hydrolysable amino acids (THAA). Both free and bound AAs (FAA and BAA, respectively) are included to determine total soil protein (Roberts and Jones 2008). Norleucine (Nle; 100 µL of 400 µg mL^−1^) was added as an internal standard. Hydrolysates were collected by centrifugation, dried and stored under 0.1 M HCl at −20 °C. AAs were isolated using acidified DOWEX 50WX8 200–400 mesh ion exchange resin (Metges and Petzke 1997) and derivatised to *N*-acetyl, *O*-isopropyl (NAIP) derivatives (Corr et al. 2007a; Knowles et al. 2010).

### Instrumental analyses

#### Total C and N analyses and ^15^N isotopic composition determinations

Finely ground soil, shoots and roots were sealed into tin capsules (50 to 70 µg N). Percentage TC, TN and ^15^N enrichment of soil, shoots and roots were determined at Rothamsted Research (North Wyke) using an elemental analyser (EA; Carlo Erba CN NA2000 analyser; Milan, Italy) coupled to a SerCon 20–22 isotope ratio mass spectrometer (IRMS; SerCon Ltd, Crewe, UK). Instrument performance was monitored, and values calibrated using standards detailed in Table S2. For consistency, all values are reported as atom %, due to high ^15^N enrichment above range of the delta scale (Brand and Coplen 2012).

#### AA quantification

A GC-FID (7890B Agilent Technologies) fitted with a DB-35 capillary column (60 m × 0.32 mm i.d., 0.5 µm phase thickness) was used for AA quantification by comparison to the internal standard. NAIP-AAs were identified based on their known elution order and comparison with AA standards (Corr et al. 2007b; Charteris et al. 2016). The carrier gas was helium (constant flow, 2.0 mL min^−1^) and the initial temperature programme was 70 °C (2 min) ramped to 150 °C (15 °C min^−1^), then to 210 °C (2 °C min^−1^) and finally to 270 °C (5 min, 8 °C min^−1^). Data were acquired and analysed using HP Chemstation (version 1.0, Agilent Technologies).

#### ^15^N enrichment of AAs

The δ^15^N values of individual AAs, as NAIP derivatives, were determined using GC-C-IRMS as outlined in Charteris et al. (2016), except the oxidation reactor comprised high purity copper and nickel wires, and was maintained at a temperature of 1030 °C. AA δ^15^N values were determined relative to that of a monitoring N_2_ gas with a known δ^15^N value. δ^15^N values were only accepted when 75% of an in-house mixture of AA standards, analysed before and after, yielded δ^15^N values within ± 1 ‰ of the known value (determined offline by EA-IRMS) and the remainder were within ± 1.5 ‰. Data were acquired and analysed using Isodat 3.0 (Thermo Scientific).

### Calculations

The percentage ^15^N retention of the applied ^15^N-label reflects the ^15^N enrichment, and the size of the N pool, and allows comparison between treatments. The enrichment (E) of a pool can be expressed as the number of moles of ^15^N derived from the applied ^15^N-substrate present in that pool (${n}_{N}$ is the number of moles of N in the pool and AFE is the atom fraction excess):

1 $$\mathrm{E}={\mathrm{n}}_{\mathrm{N}}\times \mathrm{AFE}$$

2 $$AFE={AF}_{Sample}-{AF}_{Control}$$

The atom fraction is the proportion of ^15^N in the total N pool calculated from the atom % ^15^N for bulk soil and plants, and from δ^15^N values for total hydrolysable amino acids:

3 $$AF= \frac{{R}_{std}\left(\frac{{\delta }^{15}N}{1000}+1\right)}{1+ \left(\frac{{\delta }^{15}N}{1000}+1\right)}$$

where R_std_ is the ^15^N/^14^N ratio of Air, the international isotopic standard for N (Mariotti 1983). Atom %^15^N was used for highly enriched pools, while the delta scale was required for amino acids due to lower ^15^N enrichment. This was essential to avoid loss of sensitivity of compound-specific determinations. As atom fractions, and subsequently calculated %^15^N retention/incorporation are utilised, there is not direct comparison of the two scales of ^15^N enrichment. Using the E of a pool, it is possible to calculate percentage ^15^N retention of the applied substrate using Eq. 4 (E_A_ is the number of moles of excess ^15^N applied, above natural abundance):

4 $${\% }^{15}N retention=\left(\frac{E}{{E}_{A}}\right)\times 100$$

Percentage ^15^N incorporated of applied (%^15^N_A_) and of retained (%^15^N_R_) into the soil protein pool was calculated from the concentration and δ^15^N values of individual AAs as in Charteris et al. (2016). %^15^N_A_ incorporated into AAs of the applied ^15^N was used to account for the importance of all pathways of applied ^15^N, whilst %^15^N_R_ for AAs in the soil demonstrates the ability of the soil microbial community to utilise transferred ^15^N.

Calculation of N transfer between plants was modified from Ledgard et al. (1985) to calculate the proportion of N transferred from the donor (D) to the receiver plant (R). The proportion transferred (P_transfer_) was calculated using:

5 $${P}_{transfer}= \frac{{}^{15}{N}_{R}}{{}^{15}{N}_{R}+{}^{15}{N}_{D}+{}^{15}{N}_{S}}$$

where ^15^N indicates the ^15^N content of the receiver, donor and soil (S). N transfer to the soil protein pool was also calculated in the same way using the ^15^N content of the THAA pool. The ^15^N content of the donor roots in the T_ra_C was used to calculate P_transfer_ as the ^15^N label was not evenly distributed through the plant and this was considered more representative of the ^15^N available for transfer. The ^15^N content of the soil was also included in this calculation to avoid overestimation of N transfer to the receiver plant as significant transfer to soil was observed. The ^15^N content of the pools was calculated using:

6 $${}^{15}{\mathrm{N}}_{\mathrm{X}}=\mathrm{atom }{\mathrm{\% }}^{15}\mathrm{N }{\mathrm{excess}}_{\mathrm{X}}\times {\mathrm{TN}}_{\mathrm{X}}$$

where the atom % ^15^N ${\mathrm{excess}}_{\mathrm{X}}$ is the difference between the atom % ^15^N of the control and the treatment for D, R or S pools, and TN_X_ is the total nitrogen content of the D, R and S pools. The N_transfer_ can subsequently be calculated using:

7 $${N}_{transfer}={P}_{transfer}\times T{N}_{D}$$

Finally, the percentage of N in the receiver plant derived from donor transfer (Ndft_R_) is calculated using:

8 $${\mathrm{Ndft}}_{\mathrm{R}}= \frac{{\mathrm{N}}_{\mathrm{transfer}}}{{\mathrm{TN}}_{\mathrm{R}}} \times 100$$

## Statistical analysis

All statistical analyses were performed using R statistical software (R Core Team 2021). Prior to statistical analyses, data was tested for normality (Shapiro–Wilk) and homogeneity of variance (Brown-Forsythe). Statistical analyses to determine significant differences in observations were applied throughout the study using one-way ANOVA or student t-tests. Following ANOVA analyses, when the null hypothesis was rejected, a Holm-Sidak test was used to conduct pair-wise multiple comparisons. The significance level was set at *p* ≤ 0.05 for all statistical analyses.

## Results

### One- and two compartment N transfer experiments

#### Effect of treatment on biomass 

The biomass (dry weight) of clover and ryegrass for treatments in the one- and two-compartment experiments is shown in Table 2. Clover shoot biomass was significantly less than that of the control for the ^15^N-urea treatment (*p* < 0.01), however, there was no significant difference for all other treatments. Overall, total clover biomass did not significantly vary between the treatments (*p* > 0.05). Clover root biomass was significantly larger in the ^15^N-urea only and exudation treatments compared to the control (*p* < 0.01), although these treatments were not significantly different to all other treatments. Ryegrass biomass was significantly larger compared to the control in the ^15^N-urea treatment, exudation treatment and sterile treatment (*p* < 0.05), and was also larger in all other treatments, although this difference was not significant.

**Table 2:** Biomass harvested after 480 h from split root labelling and treatments in the one- and two-compartment N transfer experiments from clover to grass. Values are mean ± SE (*n* = 4). Lower case letters indicate a significant difference between the treatments determined via a one-way ANOVA and multiple pairwise comparisons (*p* < 0.05). L_ab_C = labelling compartment, T_ra_C = transfer compartment

	Dry matter (mg plant^−1^)						
	CLOVER				RYEGRASS		
	Roots L_ab_C	Shoots	Roots T_ra_C	Total	Roots T_ra_C	Shoots	Total
Control	99 ± 25.8	341 ± 25.8 a	90 ± 13.7	530 ± 26.0	127 ± 25.8 a	318 ± 28	446 ± 41 a
^15^N-urea	88 ± 19.6	228 ± 19.6 b	111 ± 35.9	426 ± 48.4	435 ± 65.2 b	380 ± 44	801 ± 90 b
Decomposition	n/a	n/a	n/a	n/a	252 ± 30.6 ab	457 ± 42	708 ± 60 ab
Exudation	n/a	n/a	n/a	n/a	446 ± 172 b	515 ± 51	961 ± 150 b
Defoliation	35.3 ± 4.48	n/a	67.4 ± 11.9	305 ± 27.0	265 ± 45.6 ab	428 ± 59	693 ± 100 ab
Sterile	87 ± 21.7	220 ± 57.3 b	56 ± 22.8	349 ± 54.4	346 ± 101.ab	470 ± 72	816 ± 90.2 b
Fungi	74 ± 21.4	291 ± 23.1 ab	118 ± 17.6	484 ± 21.2	163 ± 26.7 ab	420 ± 62	584 ± 86 ab
Weevil	106 ± 46.2	284 ± 49.0 ab	72 ± 16.6	463 ± 71.7	188 ± 63.2 ab	320 ± 37	508 ± 80 ab
ANOVA	*p* > 0.05	*p* < 0.05	*p* > 0.05	*p* > 0.05	*p* < 0.01	*p* > 0.05	*p* < 0.001

The abundance of root nodules was also affected by treatment, with a reduced abundance in L_ab_C roots for all treatments compared to the control, except the sterile treatment (Table S3). This reduction was significant for the ^15^N-urea only (*p* < 0.05), defoliation (*p* < 0.001) and fungi (*p* < 0.05) treatments. Nodule abundance was also significantly reduced in the T_ra_C for the sterile treatment compared to the control treatment (*p* < 0.05), however, there were no other significant differences in nodules abundance in all other treatments (Table S3).

#### Percentage ^15^N retention

Percentage of the applied ^15^N-urea retained in the different pools is shown in Table 3, calculated from δ^15^N values and N content of pools (Table S4). The ‘unknown’ pool is the difference between ‘accounted for’ ^15^N and total ^15^N applied, which was presumed to be in L_ab_C roots (not determined due to very high enrichment over 10 atom% ^15^N), ^15^N-labelled L_ab_C root exudates and remaining ^15^N-label in L_ab_C sand. Other losses, such as foliar ammonia release are expected to be minor given the experimental set-up (Sommer et al. 2004). ^15^N-incorporation into clover shoots did not significantly vary with treatment, with high variation between replicates (*p* > 0.05). The weevil treatment had greater ^15^N retention in clover T_ra_C roots (2.9 ± 0.7%), which was significantly larger than the sterile treatment (*p* < 0.05), however, there were no other significant differences between treatments, due to high variation between replicates. The decomposition treatment had significantly higher ^15^N retention in T_ra_C soil compared to all other treatments (Table 3; all *p* < 0.001). This was also observed for ryegrass roots and shoots in this treatment compared to all other treatments, which were not significantly different to each other (*p* > 0.05). The variable ^15^N retention of clover indicated that the quantity of ^15^N available for transfer to the soil microbial community and ryegrass varied between treatments and replicates, which was accounted for in subsequent N transfer calculations.

**Table 3:** Percentage ^15^N retention of applied ^15^N-urea into pools. Brackets indicate the pools which were either produced separately (e.g., labelled ^15^N clover and exudates in the decomposition and exudate treatments, respectively) or pools which were removed from the experiment as a treatment (e.g., defoliation). The values were excluded from analysis due to different experimental design, but are included within the table for completeness. Pools with a * were incorporated into the T_ra_C of the treatment. Values are mean ± SE (*n* = 4). Lower case letters indicate a significant difference between the treatments determined using one-way ANOVA and multiple pairwise comparisons (*p* < 0.05). L_ab_C = labelling compartment, T_ra_C = transfer compartment

Treatment	Clover		Soil T_ra_C	Grass		Total	Unknown
	Shoots	Roots T_ra_C		Roots T_ra_C	Shoots		
^15^N-urea	37 ± 2.5	1.8 ± 0.4 ab	0.69 ± 0.11 a	0.34 ± 0.09 a	0.14 ± 0.01 a	40 ± 3.1 ab	60 ± 3.1
Decomposition	(40 ± 0.2*)	(0.33 ± 0.07)	6.8 ± 0.8 b	1.9 ± 0.5 b	4.0 ± 0.6 b	53 ± 2.1 b	47 ± 2.1
Exudation	(29 ± 5.0*)	(1.2 ± 0.3)	0.40 ± 0.09 a	0.05 ± 0.01 a	0.09 ± 0.02 a	30 ± 5.5 ab	70 ± 5.5
Defoliation	(23 ± 6.2)	0.57 ± 0.15 ab	0.67 ± 0.10 a	0.16 ± 0.04 a	0.08 ± 0.02 a	24 ± 6.5 a	76 ± 6.5
Sterile	24 ± 8.4	0.47 ± 0.26 a	0.53 ± 0.09 a	0.36 ± 0.08 a	0.38 ± 0.06 a	25 ± 7.9 a	75 ± 7.9
Fungi	22 ± 6.7	0.84 ± 0.13 ab	0.39 ± 0.07 a	0.09 ± 0.02 a	0.17 ± 0.05 a	24 ± 7.0 a	76 ± 7.0
Weevil	25 ± 6.5	2.9 ± 0.7 b	0.51 ± 0.14 a	0.28 ± 0.07 a	0.07 ± 0.01 a	29 ± 7.4 ab	71 ± 7.4
ANOVA	*p* > 0.05	*p* < 0.5	*p* < 0.001	*p* < 0.001	*p* < 0.001	*p* < 0.01	

#### Plant nitrogen transfer

**Fig. 2:**
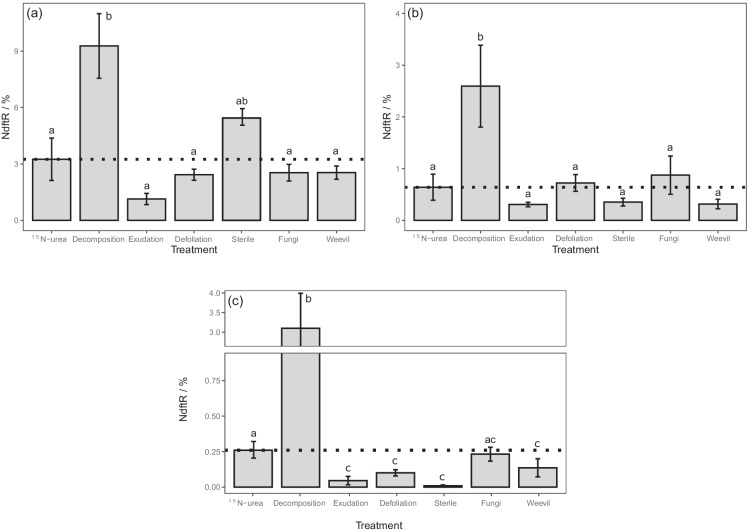
The proportion of nitrogen in (**a**) receiver plant, (**b**) soil and (**c**) soil protein pool derived from the donor plant via N transfer (Ndft_R_). Values are mean ± SE (*n* = 4). Lower case letters indicate if there are significant differences between the treatments determined via a one-way ANOVA and multiple pairwise comparisons (*p* < 0.05)

The proportion of N in the receiver plant derived from the donor plant indicated the potential for N-transfer in the different treatments. ^15^N enrichment of T_ra_C roots was used to quantify N transfer in all treatments except the decomposition treatment, where clover shoot ^15^N-enrichment was used. N transfer was greatest for the decomposition treatment, with 9.3 ± 1.7% of ryegrass-N derived from the clover shoots incorporated into the soil, which was significantly larger than all other treatments except the sterile treatment (Fig. 2a). In the sterile treatment, N transfer from clover roots accounted for 6.0 ± 0.8% of ryegrass N, while N transfer was comparable for ^15^N-urea only (3.2 ± 1.1%) and defoliation (2.4 ± 0.3%) treatments and following fungi (2.5 ± 0.5%) and weevil (2.5 ± 0.3%) addition (Fig. 2a). N transfer was least in the exudation treatment, with N transferred from the clover roots accounting for 1.1 ± 0.3% of grass N.

#### Soil N transfer

There was no observable change in the TN of soil for all treatments compared to the control and ^15^N-urea control (Table S4). The proportion of soil N derived from clover is shown in Fig. 2b, which was significantly higher for the decomposition treatment compared to all other treatments (*p* < 0.001). N transfer to soil was also higher compared to ^15^N-urea only for the defoliation (0.72 ± 0.16%) and fungi (0.88 ± 0.31%) treatments, although the difference was not significant (*p* > 0.05). The exudation, sterile and weevil treatments were comparable to the positive control (Fig. 2b).

The soil protein pool indicates newly biosynthesised AAs by the soil microbial community and the proportion of clover derived-N present in the soil protein pools is shown in Fig. 2c. This was determined via soil AA concentrations (Table S5) and compound-specific δ^15^N determination of AAs (Table S6). The THAA concentration in T_ra_C soil did not significantly vary between treatments (Table 4; *p* > 0.05), although there were some significant differences for individual AAs in the defoliation treatment compared to the control (e.g. valine (Val), leucine (Leu) and threonine (Thr), Table S5). N transfer of clover-derived ^15^N to the soil protein pool (Fig. 2) showed significant differences between treatments (*p* < 0.001). The proportion of N in the soil protein pool derived from clover was significantly higher in the decomposition treatment (1.71 ± 0.58%) compared to all other treatments (*p* < 0.001). It should be noted this may be an overestimation of newly biosynthesised THAAs derived from clover-^15^N as it was not possible to separate partly decomposed clover in this treatment. The ^15^N-urea treatment was also significantly higher than all treatments (excluding decomposition; 0.0046 ± 0.003%, *p* < 0.01) and all other treatments were not significantly different from each other (*p* > 0.05). This indicated that whilst N transfer to soil did not significantly vary, assimilation into the microbial protein pool was reduced by all treatments except decomposition, compared to the positive control. Within the THAA pool, the distribution of ^15^N derived from clover did not significantly vary between treatments, with ^15^N incorporation dominated by glutamine/glutamate (Glx), aspargine/aspartate (Asx), alanine (Ala), glycine (Gly) and proline (Pro), with comparatively low incorporation into Val, phenylalanine (Phe), hydroxyproline (Hyp) and tyrosine (Tyr) (Figure S2).

**Table 4:** N transfer to soil and THAA derived from clover in one-and two-compartment experiments. Values are mean ± SE (*n* = 4). THAA = total hydrolysable amino acids; AA = amino acids

Treatment	Soil N transfer / %	Soil amino acids	
		THAA Concentration / mg g^−1^ soil	AA N transfer / %
^15^N-urea	0.64 ± 0.25	12.6 ± 0.7	0.26 ± 0.05
Decomposition	2.59 ± 0.76	13.0 ± 1.8	1.79 ± 0.52
Exudation	0.31 ± 0.04	12.3 ± 2.7	0.045 ± 0.03
Defoliation	0.72 ± 0.16	8.2 ± 1.6	0.10 ± 0.02
Sterile	0.35 ± 0.07	10.9 ± 1.3	0.018 ± 0.005
Fungi	0.88 ± 0.31	10.5 ± 1.8	0.22 ± 0.07
Weevil	0.32 ± 0.09	11.9 ± 2.0	0.14 ± 0.06

### Three-compartment N transfer experiments

#### Biomass 

Addition of ^15^N-urea significantly increased clover biomass produced in the clover to ryegrass experiment compared to controls, likely due to a priming effect induced by higher N availability in the L_ab_C (Table 5a). In the reverse experiment, ryegrass to clover, ryegrass shoot biomass increased, however, this was not significant (*p* > 0.05) (Table 5b). In the T_ra_C, in both experiments, the total biomass of the receiver plant was low in the ^15^N-urea treatment compared to the control treatment, although the observed differences were not significant (*p* > 0.05).

**Table 5:** Biomass harvested at 100 h from split root labelling in reverse transfer of N between (a) clover and ryegrass and (b) ryegrass and clover. Values are mean ± SE (*n* = 4). L_ab_C = labelling compartment, T_ra_C = transfer compartment, R_ec_C = receiving compartment

	Dry matter / mg plant^−1^					
	Donor plant			Receiver plant		
(a)	Clover			Ryegrass		
	Roots L_ab_C	Shoots	Roots T_ra_C	Roots T_ra_C	Shoots	Roots R_ec_C
Control	20 ± 3.2	101 ± 22	22 ± 7.7	35 ± 8.3	344 ± 57	38 ± 8.2
^15^N-urea	39 ± 4.4	190 ± 19	38 ± 7.0	19 ± 12	317 ± 21	40 ± 7.8
t-test	*p* < 0.01	*p* < 0.05	*p* > 0.05	*p* > 0.05	*p* > 0.05	*p* > 0.05
(b)	Ryegrass			Clover		
	Roots L_ab_C	Shoots	Roots T_ra_C	Roots T_ra_C	Shoots	Roots R_ec_C
Control	35 ± 31	220 ± 27	26 ± 8.8	20 ± 4.1	153 ± 22	22 ± 5.7
^15^N-urea	17 ± 11	289 ± 67	58 ± 17	15 ± 5.0	114 ± 25	16 ± 10
t-test	*p* > 0.05	*p* > 0.05	*p* > 0.05	*p* > 0.05	*p* > 0.05	*p* > 0.05

#### Root exudation

**Fig. 3:**
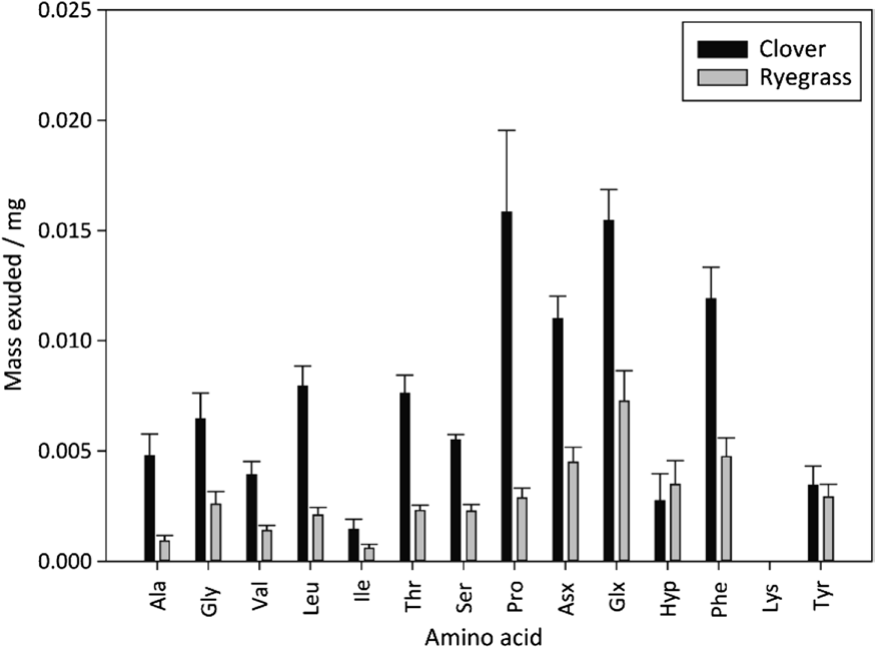
Mass of individual AAs exuded from clover (black) and ryegrass (grey) roots. Values are mean ± SE (*n* = 4) and are corrected for recovery rates of AA standards from sand (Table S7)

THAAs released from roots were significantly higher for clover (0.10 ± 0.01 mg) in the clover to ryegrass experiment compared to ryegrass in the ryegrass to clover experiment (0.041 ± 0.006 mg; *p* < 0.01). The THAA exudates account for varying recovery of individual AAs, due to retention in the sand, determined using a standard AA mixture (Table S7). Within the THAAs exuded by clover, Pro, Asx, Glx and Phe were the most abundant AAs exuded by clover and Glx, Asx, Phe and Hyp were the most abundant AAs exuded by grass roots (Fig. 3).

#### % ^15^N retention

The ^15^N retention into clover, soil and grass pools were determined from ^15^N values, %TN and plant biomass (Table S8, Tables 5 and 6). In the clover to ryegrass experiment, uptake into clover biomass accounted for 41.7 ± 4.0%, largely in R_ec_C roots and clover shoots. Only a small portion was transferred to T_ra_C roots (0.72 ± 0.11%). Comparatively higher uptake into the grass donor plant in the grass to clover experiment was observed, accounting for 72.6 ± 11.1% of applied ^15^N, with the majority of this retained ^15^N present in grass shoots. The higher ^15^N retention into donor T_ra_C roots in ryegrass to clover compared to clover to ryegrass meant higher availability for N transfer. This was supported by significantly higher ^15^N retention in the receiver plant in ryegrass to clover than clover to ryegrass (*p* < 0.001). Retention in both soil compartments was also higher in the ryegrass to clover compared to clover to ryegrass (Table 6), although enrichment in the R_ec_C was below detection limits in the clover to ryegrass experiment.

**Table 6:** % ^15^N retention into pools following N transfer from clover to grass and grass to clover 100 h after ^15^N-urea application to L_ab_C. (n.d. = not detected). Values are mean ± SE (*n* = 4). L_ab_C = labelling compartment, T_ra_C = transfer compartment, R_ec_C = receiving compartment

	Donor plant			Soil T_ra_C /%	Receiver plant			Soil R_ec_C / %	Total uptake / %
	Roots L_ab_C / %	Shoots / %	Roots T_ra_C / %		Roots T_ra_C / %	Shoots / %	Roots R_ec_C / %		
Clover to grass	18 ± 1.9	23 ± 2.0	0.72 ± 0.11	0.31 ± 0.07	0.004 ± 0.001	0.004 ± 0.001	0.002 ± 0.0006	n.d.	42.0 ± 4.1
Grass to clover	4.0 ± 0.8	67 ± 10	1.6 ± 0.3	0.52 ± 0.16	0.52 ± 0.09	0.02 ± 0.003	0.005 ± 0.002	0.21 ± 0.004	73.7 ± 11.3

#### N transfer

The proportion of donor derived-N in the receiver plant was calculated based on the yield-dependent calculation (Eq. 8), accounting for differences in available ^15^N for transfer. N transfer to ryegrass from clover was 0.8 ± 0.3% and N transfer to clover from ryegrass was 1.3 ± 0.6% at 100 h. There was no significant difference between the two N transfer directions. Greater N transfer to the receiver plant was associated with a smaller proportion of donor-derived N in the soil at 100 h, while incorporation into the soil protein pool was comparable in both directions (Table 7). Within the soil protein pool, THAA concentration was larger for both treatments compared to the control, and in the R_ec_C compared to the T_ra_C in the same treatment (Table 7). However, there was no significant difference between the incubations for the overall THAA concentration (*p* > 0.05), although there were differences within the THAA pool for individual AAs (e.g. Phe, Lys and Tyr) (Tables S9 and S10). ^15^N assimilation into the individual AAs was calculated from δ^15^N values (Tables S11 and S12) and AA concentrations (Tables S9 and S10). Within the AA pool, ^15^N-incorporation was largest for Glx and Asx in the ryegrass to clover T_ra_C. These pools also showed higher ^15^N-incorporation in the clover to ryegrass T_ra_C, with Pro also showing high ^15^N-incorporation, which may reflect the distribution of AAs released in root exudates (Fig. 3). It was not possible to detect any assimilation into the soil protein pool or transfer to the bulk soil in the clover to ryegrass R_ec_C soil, while incorporation into individual AAs in the ryegrass to clover was dominated by Asx, Glx, serine (Ser), Ala and Gly (Figure S3).

**Table 7:** THAA concentration and soil and AA N transfer from clover to grass and grass to clover. Values are mean ± SE (*n* = 4)

N transfer direction	Treatment	Soil N transfer / %	T_ra_C	R_ec_C	T_ra_C	R_ec_C
			THAA concentration / mg g^−1^ soil		AA N transfer / %	
Clover to grass	Control	-	8.9 ± 2.3	11.8 ± 1.1	-	-
	^15^N-urea	0.083 ± 0.011	11.4 ± 0.4	13.1 ± 1.3	0.17 ± 0.09	-
Grass to clover	Control	-	10.0 ± 0.6	12.2 ± 0.7	-	-
	^15^N-urea	0.17 ± 0.06	11.7 ± 2.0	13.8 ± 1.2	0.13 ± 0.03	0.15 ± 0.09

## Discussion

**Fig. 4:**
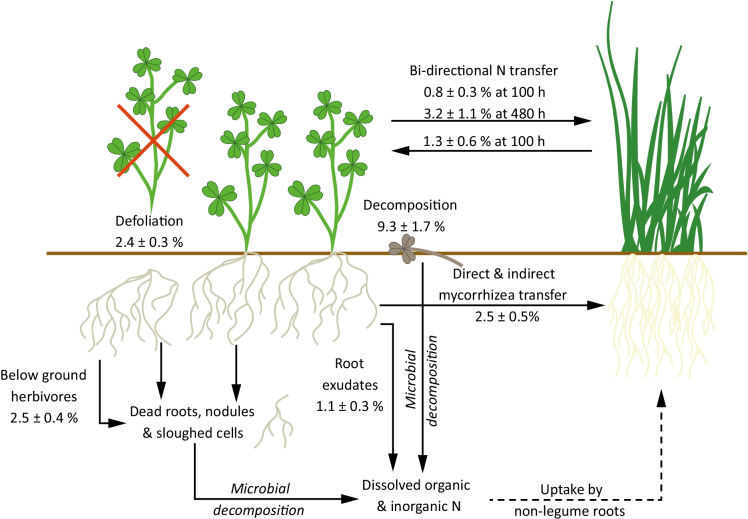
The N transfer (Ndft_R_) pathways in a model clover-ryegrass system as affected by management (simulated grazing by defoliation, N fertiliser application or shoot residue decomposition) and presence of soil organisms (weevils, mycorrhiza). The bidirectional N transfer at 100 h was determined via the three-compartment experiment. All values are mean ± SE (*n* = 4)

Optimisation of N transfer in mixed clover-ryegrass swards provides an opportunity to use natural ecosystem processes to improve nutrient supply and increase productivity in intensively managed grassland systems (Elgersma and Hassink 1997; Paynel et al. 2008; Fustec et al. 2010; Thilakarathna et al. 2016). This approach has the potential to provide both economic and environmental benefits, through reduced N fertiliser requirements. This study has investigated important N transfer pathways and their potential contribution to overall N transfer using a model clover-ryegrass system. The application of a ^15^N-labelling approach allowed us to identify and quantify the strength of N transfer via a range of interdependent pathways between white clover and perennial ryegrass (Fig. 4). The modifications to the clover-ryegrass model system aimed to quantify selected N transfer pathways (e.g. exudation), investigate possible increased N transfer via due to land management (e.g. N input, decomposition, defoliation simulating grazing) and the presence of organisms (weevils and mycorrhiza addition).

^15^N transfer from clover to ryegrass following only ^15^N-urea fertilisation was relatively minor compared to previous observations (Høgh-Jensen and Schjoerring 2000; Rasmussen et al. 2007; Gylfadóttir et al. 2007). Observed N transfer is generally low in split root labelling experiments (less than 10%) via this natural N uptake pathway (van Kessel et al. 1985; Martin et al. 1991; Ikram et al. 1994; Chalk et al. 2014). Furthermore, the relatively large variability associated with the estimated N transfer has previously been observed in N transfer quantification (Kurppa et al. 2010). A key contributing factor in this study was likely the N status of the soil, which received regular N inputs during livestock production across the farm platform, therefore, N demands were likely met by the existing soil N pool, and there was no indication of N deficiency (e.g. yellow leaves, stunted growth) (Bol et al. 2004; Harrod and Hogan 2008; Orr et al. 2016). N transfer has previously been found to be driven by a source and sink relationship between plants in low N status soils with low N inputs (Murray and Clements 1998; Gylfadóttir et al. 2007). N transfer of the same magnitude as this study has been observed in higher N status soil with other legume-non-legume systems, suggesting plant N transfer is less important in higher N status soils (Sen and Chalk 1996; Teste et al. 2015). Other factors which may have limited N transfer include the young sward age, limited plant size in incubation tubes and disrupted fungal networks during incubation set-up (Jones and Willett 2006; Louarn et al. 2015). N demands were met by increased N fixation, as decreased nodulation under N fertilisation indicated reduced N fixation requirements. Therefore, soil properties (i.e. N status) and crop maturity are important factors for consideration when utilising optimised N transfer in sustainable land management strategies to improve crop N supply. N transfer to soil and the soil protein pool in the ^15^N-urea only treatment was also relatively low compared to the receiving plant (0.64 ± 0.25% and 0.26 ± 0.10%, respectively). Within the THAA pool, incorporation into individual AAs reflected fundamental biosynthetic pathways of AA biosynthesis (Figure S2a). Incorporation was highest in Glx, due to its central role in AA biosynthesis, with NH_4_^+^ incorporated via the GS/GOGAT (glutamine synthetase/ glutamine oxoglutarate amino transferase) and GDH pathways (glutamate dehydrogenase) (Nelson and Cox 2017; Caspi et al. 2018). The Glx pool is subsequently essential for the formation of other AA pools, either by direct conversion (e.g. Pro) or transamination reactions (e.g. Ser, Phe, and Tyr) (Knowles et al. 2010; Nelson and Cox 2017; Caspi et al. 2018). AAs with more biosynthetic steps exhibited lower ^15^N incorporation (e.g. Phe and Tyr via chorismate), as did secondary AAs (e.g. Hyp) (Adams and Frank 1980). Incorporation was also influenced by pool size, with higher incorporation observed in larger pools (e.g. Ala, Gly, Pro). Hierarchical trends in ^15^N incorporation for individual amino acids were comparable for all treatments as this reflected the biosynthetic pathways of AA biosynthesis (Figure S2).

### Quantification of selected N transfer pathways

One pathway that was possible to determine in isolation was exudation, and *ca.* 35% of total N transfer from clover to ryegrass in the unmodified system (^15^N-urea only) was derived from clover root exudates. This significant contribution to total N transfer has previously been observed in young clover-ryegrass mixed swards (Paynel et al. 2001; Paynel and Cliquet 2003). N-containing root exudates are transferred via direct root-root contact, mass flow and microbial-mediated transfer in a mixed cropping system, although direct root-to-root contact was removed in this model system. The proportion of N transferred from clover to the soil microbial community by exudation accounted for 20% of the N transferred to the soil protein pool, demonstrating the importance of root exudates for microbial N acquisition and their role in indirect transfer of exuded N to non-legumes following microbial turnover (Coskun et al. 2017). N transfer via root exudates will likely be further increased when legume and non-legume roots are allowed to intermingle in soil, due to the importance of root contact in this process and enhanced exudation via microbe-plant and plant-plant interactions (Merbach et al. 1999; Inderjit and Weiner 2001; Bais et al. 2006; Canarini et al. 2019).

The microbial community is central to several N transfer pathways, and this was confirmed by ^15^N incorporation and N transfer into the soil microbial protein pool (Fig. 2 and S2). Sterilisation aimed to remove this pathway, limiting N transfer to mass flow and direct root-to-root contact. The increased N transfer observed was likely due to reduced competition between ryegrass and the soil microbial community (Inselsbacher et al. 2010). Comparable transfer to the soil was observed, which was more bioavailable to ryegrass with lower microbial activity, supported by lower incorporation into the soil protein pool. There was still evidence of assimilation into the soil protein pool, indicating recolonization of the soil by microbes originating from root surfaces, yielding an optimised microbial community, which likely also supported plant N transfer (Marschner and Rumberger 2004).

### Modification of N transfer pathways under management strategies

Management strategies, for example grazing and cover crop incorporation, were simulated in the model system to quantify changing N transfer compared to fertilisation only. Defoliation, which simulated grazing, reduced N translocations between compartments, and overall uptake into the plant soil system. Defoliation was hypothesised to result in root decomposition due to plant death, increasing clover-derived N availability and subsequently N transfer to companion plants. Rather than increasing N transfer to ryegrass through decomposition of roots after plant death, defoliation resulted in a small decrease in plant N transfer, and a significant decrease in N transfer to the microbial protein pool, indicating no significant root decomposition compared to the positive control. The reduction in N retention and N transfer reflected the reduced translocation of N by clover, coupled with higher N demands for regrowth, which was observed during the incubation (Del-Val and Crawley 2004). The capacity of clover to regrow while competing with ryegrass for nutrients has been previously observed, however, previous studies have also shown increased N transfer to ryegrass and the soil microbial community which was not observed (Del-Val and Crawley 2004; Ayres et al. 2007). This may be due to the young sward age and single defoliation, with N transfer shown to increase in older mixed swards following repeated defoliation (Høgh-Jensen and Schjoerring 1997; Jørgensen et al. 1999; Louarn et al. 2015). Therefore, changes in N transfer across the lifespan of a mixed sward should be accounted for in land management strategies aiming to maximise N transfer.

Decomposition of clover shoots was representative of turnover of above ground biomass, and residue incorporation from a cover crop. Clover N, released by decomposition, was a significant source of N for ryegrass due to favourable decomposition conditions (e.g., moisture, aerobic conditions, direct contact with soil, C:N ratio) (Weil and Brady 2017) and no plant regrowth was possible. The high N transfer (9.3 ± 1.7%) to ryegrass was supported by microbially-mediated decomposition, reflected in N transfer to the soil protein pool (1.79 ± 0.52%), indicating the central role the soil microbial community plays in N transfer via decomposition of shoot material. N transfer via this pathway was higher than root decomposition which was thought to be low in the defoliation treatment, as white clover shoots decompose faster than roots (Barel et al. 2019). Although N transfer was high, there was no significant biomass increase observed compared to the ^15^N-urea only treatments and no change in the N status of the soil, likely due to the small scale and short experimental period. Enhanced N transfer following shoot incorporation, such as from cover crops into soil, confirmed the importance of clover death and microbially-mediated decomposition, which was a major source of N to partner crops (Trannin et al. 2000). The role of cover crops in N supply to partner crops warrants further investigation, as larger scale studies have identified yield improvements for cash crops, due to N transfer from decomposition of incorporated cover crops (Ledgard 2001; Abdalla et al. 2019).

### Effect of biota on N transfer

Soil biota play an important role in N transfer, both via competition with companion plants for legume-N and supporting the transformation of N through microbially-mediated decomposition. Mycorrhizal networks are thought to play an important role in promoting N transfer via CMNs (common mycorrhizal networks) and indirect uptake and translocation (Cheng and Baumgartner 2004; Wahbi et al. 2016). This pathway was enhanced by addition of mycorrhizae, however, there was no observed increase in N transfer to ryegrass coupled with decreased biomass for both clover and ryegrass. The lack of increased N transfer via mycorrhizal networks, coupled with reduced plant biomass has also been observed in other studies investigating the importance of mycorrhizal networks for N transfer (Haystead et al. 1988; Barea et al. 1989; Hodge and Fitter 2013; Wahbi et al. 2016; Ren et al. 2017). Enhanced N transfer via mycorrhizal networks has been observed with low N availability in soils, driven by a concentration gradient through hyphal links (Jalonen et al. 2009). Therefore, the higher soil N status used in this study limited the importance of clover-derived N as an N source for ryegrass and thus N transfer pathways, such as mycorrhizal networks. N transfer to the soil protein pool was also reduced, although transfer to soil was increased compared to the positive control. Although it was not possible to confirm the extent of mycorrhizal colonisation, it is suggested the addition of the mycorrhizal fungi may have disrupted the existing plant and microbial communities, reducing clover-derived ^15^N transfer to ryegrass via microbial pathways, and assimilation into the soil protein pool.

Clover pests, such as weevils, reduce resilience to climatic factors and infection while also increasing N transfer, by reducing the N acquisition ability of clover roots, sloughing of roots and damage to root cells (Gerard et al. 2007). This was not observed in the model system following infestation with weevil eggs, with lower N transfer to ryegrass and the soil microbial community. It is suggested any damage to roots and nodules increased N demand by the clover to repair damage, which was also observed following defoliation. This increased competition for available ^15^N thus reduced the availability of N for transfer to ryegrass and the soil microbial community. This was supported by increased ^15^N retention in clover T_ra_C roots, suggesting re-uptake of exuded N to repair damaged roots and nodules. Furthermore, there was limited damage observed to roots and nodules, and no change in clover biomass (Goldson and Jamieson 1988; Murray and Hatch 1994; Murray et al. 2002; Gerard et al. 2007). Therefore, the length of infestation may have limited the damage to clover root, with hatching time observed to be over 11 days at 25 ºC (Johnson et al. 2010) and increased damage to clover observed in subsequent years after infestation (Gerard et al. 2007).

### Reverse N transfer

N transfer from clover to ryegrass rather than the reverse direction has previously been shown to dominate in mixed swards (Høgh-Jensen and Schjoerring 2000; Rasmussen et al. 2007; Chalk et al. 2014). It was surprising to observe that N transfer from ryegrass to clover was higher than clover to ryegrass (over 100 h). The dominance of N transfer from ryegrass to clover has not been previously observed, although comparable transfer in the two directions has been observed in mixed cropping systems (Jamont et al. 2013). The higher N transfer from ryegrass to clover, and also to the soil and soil microbial community (in the R_ec_C), reflected higher availability of ^15^N in ryegrass donor T_ra_C roots, ultimately meaning more ^15^N was available for transfer and higher root biomass for the ryegrass donor plant. The ryegrass was more efficient, or had higher N demand, compared to the clover at utilising the applied ^15^N, as indicated by higher ^15^N retention, higher biomass and transfer of ^15^N from the L_ab_C through to T_ra_C roots and soil. Clover may have also utilised unlabelled N via N_2_ fixation to meet N demand, although decreased nodulation observed compared to the control experiment does not support this, and N fixation was likely suppressed with N application. Therefore, while ryegrass root exudation was lower than clover, it more quickly reflected the provided ^15^N source, due to higher ^15^N-enrichment of ryegrass donor roots, influencing the higher ^15^N transfer from ryegrass to clover in this model system. This interesting observation in this model system should be further investigated to consider whether sward age or relatively available soil N, shown to reduce N transfer from clover in this study, may also influence the ability of ryegrass to act as an N source in a mixed sward (Del-Val and Crawley 2004; Ayres et al. 2007).

### Conclusions

N transfer from clover to ryegrass has the potential to reduce fertiliser requirements and provide economic and environmental benefits. This study used a model system to investigate specific N transfer pathways and the effect of land management on the magnitude of N transfer in a mixed clover-ryegrass pasture. Key findings were:

(i) N transfer was relatively low in these model system studies using a soil from a grassland pasture with a history of frequent fertiliser application, compared to previous observations of N transfer in a clover-ryegrass pasture. Of total N transfer, 35% was derived via root exudation.
(ii) The microbial community played an important role in N transfer in all treatments, via assimilation of clover derived ^15^N into the soil protein pool, and subsequent release for plant N supply. This confirms the utility of compound-specific ^15^N-SIP to elucidate the role of microbial N-cycling in this setting.
(iii) Factors including previous management history and sward age must be considered when assessing the potential for N transfer. Low N transfer was observed overall due to previous N additions, and N transfer was also low following a single defoliation in this young sward. It is suggested this would increase with repeated defoliation and sward age.
(iv) Decomposition of clover shoots significantly increased N transfer from clover to ryegrass, representing the use of clover as a cover crop and subsequent incorporation into soil.
(v) Damage to clover (e.g. defoliation and weevils) reduced N transfer to the soil protein pool, likely due to N requirements for repair of clover roots and shoots, with plants out-competing the soil microbial community for transferred N.
(vi) N transfer was bi-directional, with higher N transfer from ryegrass to clover observed on a short time scale in this model system as ryegrass was more efficient at distributing incorporated N to different portions of the plant.

Overall, quantifying the individual mechanisms for N transfer from clover to ryegrass has confirmed the relative role of N transfer pathways, particularly the role of the soil microbial community. Future studies should extend this mechanistic level of detail regarding N transfer to field settings, including quantifying N transfer as a function of soil N status, sward age and stocking density.

## Supplementary Information

Below is the link to the electronic supplementary material.

Supplementary file1 (DOCX 545 KB)
