# Supplementary material for: Mechanisms of nitrogen transfer in a model clover-ryegrass pasture: a 15N-tracer approach
Source: Plant Soil. 2022 Jul 28;480(1-2):369–89. doi: 10.1007/s11104-022-05585-0 (PMC9705487; doi:10.1007/s11104-022-05585-0)
Supplement: Supplementary file 1 — Supplementary file1 (DOCX 545 KB) [file 11104_2022_5585_MOESM1_ESM.docx]

Mechanisms of nitrogen transfer in a model clover-ryegrass pasture: a ^15^N-tracer approach

Michaela K. Reay^a^, Katrina A. Pears^a^, Alison Kuhl^a^, Richard P. Evershed^a^, Phillip J. Murray^b†^, Laura M. Cardenas^b^, Jennifer A. J. Dungait^b#^, and Ian D. Bull^a *^

1. Organic Geochemistry Unit, School of Chemistry, University of Bristol, Cantock’s Close, Bristol, BS8 1TS
2. Department of Sustainable Agriculture Sciences, Rothamsted Research- North Wyke, Okehampton, Devon, EX20 2SB

# Current address: Carbon Management Center, SRUC - Scotland’s Rural College, Edinburgh, Scotland, UK, EH9 3JG; and Geography, CLES - Amory Building. University of Exeter, Exeter, UK, EX4 4RJ.

^†^ School of Agriculture, Food and Environment, Royal Agricultural University, Cirencester, GL7 6JS

* *Corresponding author: Ian D. Bull, ian.d.bull@bristol.ac.uk*

Figure S 1: Georeferenced location of soil sampling sites. A total of 264 soil cores (10 cm depth, 5.5 cm diameter) were collected from the North Wyke Farm Platform (50°46’N, 3°54’W). Soil was collected on a 50 m grid, with geo-referenced points located using a handheld Trimble GPS unit.

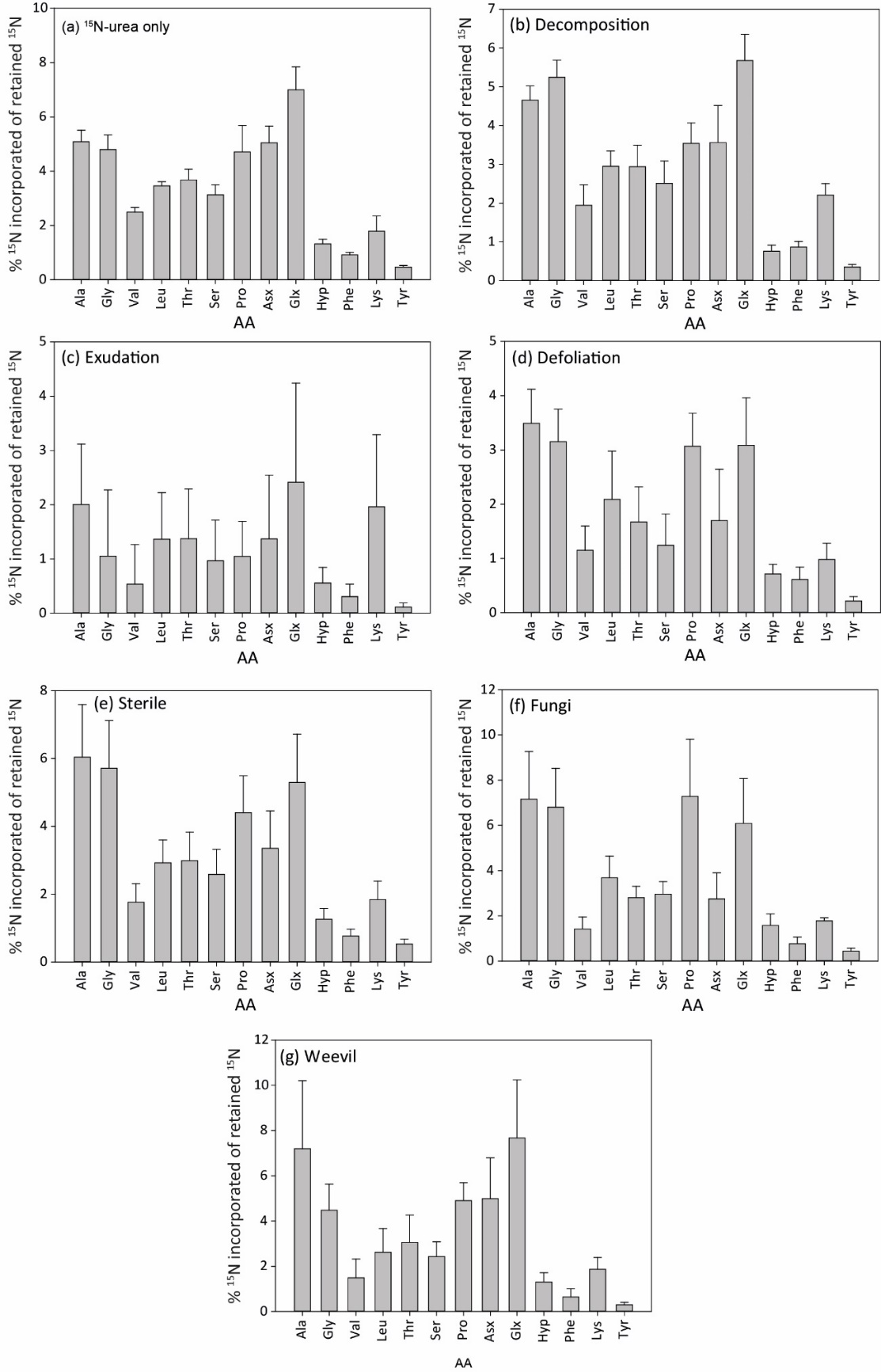


Figure S 2: Percentage ^15^N incorporation of retained ^15^N into individual soil amino acids. Values are mean (*n* = 4).


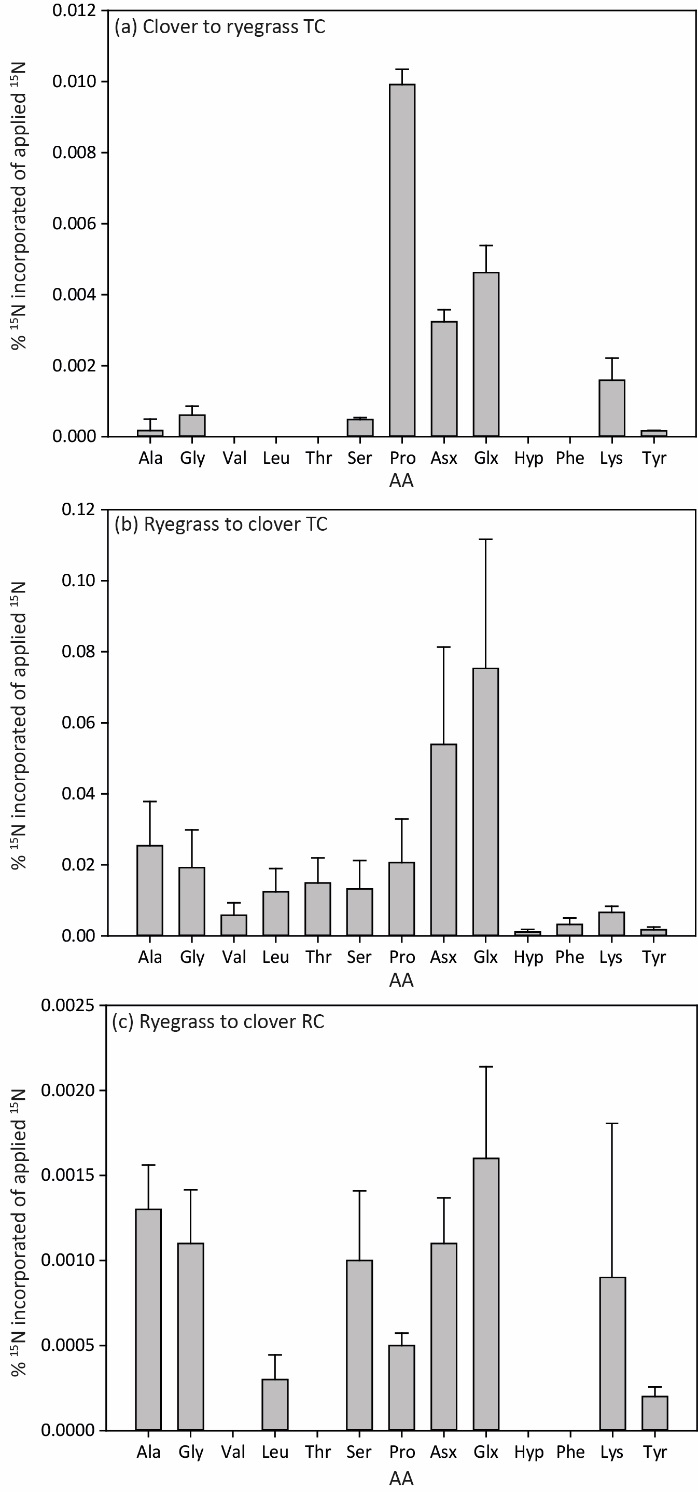


Figure S 3: Percentage ^15^N incorporation of applied ^15^N into individual soil amino acids in the three compartment (a) clover to ryegrass transfer compartment soil, (b) ryegrass to clover transfer compartment soil and (c) ryegrass to clover receiving compartment soil. Clover to ryegrass RC soil is not presented as there was not observable ^15^N incorporation in this compartment. Values are mean (*n* = 4).

Table S 1: The modified nutrient solution, adapted from the Arnon’s solution detailed in Hewitt (1966) used to water mother plans and incubations. N was omitted to promote nodulation in the mother clover plant and to ensure only one source of N was added in the incubation experiments. The modified nutrient solution at one fifth strength was used to water mother clover and ryegrass plants weekly and the modified nutrient solution at full strength was used in incubation experiments.

|  |  | Concentration full strength nutrient solution / mg l^-1^ | Concentration one-fifth strength nutrient solution / mg l^-1^ |
| --- | --- | --- | --- |
| Macro-nutrients | NH_4_NO_3_ | 572 | 114 |
|  | K_2_SO_4_ | 446 | 89.2 |
|  | Ca(H_2_PO_4_)_2_.4H_2_O | 126 | 25.2 |
|  | MgSO_4_.7H_2_O | 493 | 98.6 |
| Trace elements | H_3_BO_4_ | 2.86 | 0.572 |
|  | MnCl_2_.4H_2_O | 1.81 | 0.362 |
|  | CuSO_4_.5H_2_O | 0.08 | 0.016 |
|  | ZnSO_4_.7H_2_O | 0.22 | 0.044 |
|  | H_2_MoO_4_ | 0.09 | 0.018 |
| Iron | FeSO_4_.7H_2_O | 7.47 | 1.49 |
|  | H_2_SO_4_ (97 %) | 0.25 | 0.05 |

Table S 2: Standards used to monitor instrument performance and calibration of determined %TN and ^15^N values from EA-IRMS analysed (Section 2.4.1).

| Standard | % N | % C | ^15^N value | δ^13^C (δ_VPDB_) / ‰ |
| --- | --- | --- | --- | --- |
| IA R001 (flour) | 1.88 | 40.20 | 2.55 ‰ | -26.43 |
| AQC 206 (flour) | 1.78 | 41.57 | 2.76 ‰ | -25.80 |
| AQC 38 (soil) | 0.22 | 5.54 | 251.9 ‰ | -25.68 |
| AQC 209 (grass) | 1.95 | 42.76 | 2.79 atom %^15^N | -28.60 |

Table S 3: Number of root nodules per gram of roots (dry weight). Root nodule numbers were normalised to mass of dry root collected from each compartment.

|  | RC / root nodules g^-1^ roots | | TC / root nodules g^-1^ roots | |
| --- | --- | --- | --- | --- |
|  | Mean | SEM | Mean | SEM |
| Control | 343 a | 43 | 308 a | 38 |
| ^15^N-urea | 199 b | 25 | 289 ab | 36 |
| Decomposition | n/a | n/a | n/a | n/a |
| Exudation | 226 ab | 30 | 295 ab | 34 |
| Defoliation | 25 c | 4.5 | 147 ab | 26 |
| Sterile | 357 a | 33 | 131 b | 12 |
| Fungi | 175 bc | 12 | 306 ab | 26 |
| Weevils | 285 ab | 43 | 253 ab | 33 |
|  |  |  |  |  |
| ANOVA | <0.001 | | <0.01 | |

Table S 4: Percentage total nitrogen and ^15^N enrichment as atom% ^15^N for in modified N transfer incubation experiments. Values are mean (*n* = 4).

| Treatment | Clover shoots | | | | Clover roots TC | | | | | | Soil TC | | | | Grass roots TC | | | | | Grass shoots | | | | | |
| --- | --- | --- | --- | --- | --- | --- | --- | --- | --- | --- | --- | --- | --- | --- | --- | --- | --- | --- | --- | --- | --- | --- | --- | --- | --- |
|  | % TN | SE | Atom % ^15^N /% | SE | | % TN | SE | Atom % ^15^N /% | SE | % TN | | SE | Atom % ^15^N /% | SE | | % TN | SE | Atom % ^15^N /% | SE | | % TN | SE | Atom % ^15^N /% | SE |  |
| Control | 2.53 | 0.29 | 0.37 | 0.01 | | 1.94 | 0.07 | 0.37 | 0.01 | 0.50 | | 0.005 | 0.369 | 0.002 | | 0.96 | 0.09 | 0.368 | 0.001 | | 1.26 | 0.09 | 0.368 | 0.001 |  |
| ^15^N-urea only | 2.70 | 0.27 | 3.58 | 2.05 | | 1.97 | 0.20 | 1.08 | 0.44 | 0.49 | | 0.004 | 0.380 | 0.011 | | 0.96 | 0.08 | 0.501 | 0.087 | | 1.36 | 0.08 | 0.396 | 0.024 |  |
| Decomposition | 3.34 | 0.03 | 2.19 | 1.80 | | 1.96 | 0.07 | 0.47 | 0.07 | 0.48 | | 0.004 | 0.445 | 0.070 | | 1.49 | 0.04 | 0.808 | 0.313 | | 2.42 | 0.04 | 0.680 | 0.247 |  |
| Exudation | 2.84 | 0.11 | 3.19 | 2.18 | | 1.84 | 0.22 | 1.07 | 0.42 | 0.50 | | 0.009 | 0.372 | 0.004 | | 0.86 | 0.05 | 0.377 | 0.008 | | 1.37 | 0.05 | 0.378 | 0.008 |  |
| Defoliation | 2.89 | 0.21 | 3.37 | 2.00 | | 1.87 | 0.23 | 0.77 | 0.28 | 0.50 | | 0.004 | 0.375 | 0.008 | | 1.00 | 0.09 | 0.416 | 0.039 | | 1.22 | 0.09 | 0.380 | 0.010 |  |
| Sterile | 2.58 | 0.19 | 3.98 | 2.18 | | 2.15 | 0.19 | 0.82 | 0.34 | 0.45 | | 0.008 | 0.375 | 0.006 | | 1.37 | 0.26 | 0.441 | 0.030 | | 2.11 | 0.22 | 0.407 | 0.032 |  |
| Fungi | 3.06 | 0.03 | 2.60 | 1.39 | | 2.35 | 0.06 | 0.71 | 0.23 | 0.47 | | 0.009 | 0.373 | 0.005 | | 0.97 | 0.04 | 0.420 | 0.033 | | 1.92 | 0.13 | 0.385 | 0.005 |  |
| Weevil | 2.65 | 0.07 | 3.43 | 1.97 | | 2.03 | 0.14 | 1.65 | 0.60 | 0.46 | | 0.004 | 0.374 | 0.005 | | 1.06 | 0.09 | 0.499 | 0.066 | | 1.79 | 0.20 | 0.379 | 0.001 |  |

Table S 5: Concentration of individual amino acids in soil. Lower case letters indicate significant differences in the concentration of individual amino acids following One0way ANOVA and multiple pairwise comparison (Holm-Sidak, P<0.05). Values are mean (*n* = 4).

|  | Concentration / mg g^-1^ | | | | | | | | | | | | | | | | P |
| --- | --- | --- | --- | --- | --- | --- | --- | --- | --- | --- | --- | --- | --- | --- | --- | --- | --- |
|  | Control | | ^15^N-urea only | | Decomposition | | Exudation | | Defoliation | | Sterile | | Fungi | | Weevil | |  |
|  | Mean | SEM | Mean | SEM | Mean | SEM | Mean | SEM | Mean | SEM | Mean | SEM | Mean | SEM | Mean | SEM |  |
| Ala | 1.41 | 0.05 | 1.23 | 0.04 | 1.40 | 0.19 | 1.25 | 0.01 | 1.03 | 0.05 | 1.28 | 0.11 | 1.41 | 0.17 | 1.36 | 0.14 | ns |
| Gly | 1.49 | 0.06 | 1.31 | 0.06 | 1.45 | 0.16 | 1.36 | 0.01 | 1.09 | 0.04 | 1.37 | 0.11 | 1.59 | 0.18 | 1.44 | 0.17 | ns |
| Val | 1.00 a | 0.04 | 0.85 ab | 0.02 | 0.79 ab | 0.13 | 0.75 ab | 0.17 | 0.47 b | 0.12 | 0.64 ab | 0.09 | 0.53 b | 0.05 | 0.66 ab | 0.09 | >0.001 |
| Leu | 0.86 a | 0.02 | 0.79 ab | 0.02 | 0.92 a | 0.05 | 0.79 ab | 0.04 | 0.63 b | 0.07 | 0.78 ab | 0.03 | 0.72 ab | 0.10 | 0.75 ab | 0.04 | >0.01 |
| Thr | 1.58 a | 0.10 | 1.38 ab | 0.10 | 1.28 ab | 0.19 | 1.16 ab | 0.40 | 0.67 b | 0.24 | 0.93 ab | 0.15 | 0.70 ab | 0.11 | 1.15 ab | 0.28 | >0.05 |
| Ser | 0.98 | 0.06 | 0.88 | 0.05 | 0.77 | 0.12 | 0.75 | 0.26 | 0.41 | 0.16 | 0.65 | 0.11 | 0.69 | 0.13 | 0.78 | 0.20 | ns |
| Pro | 1.09 | 0.04 | 0.94 | 0.03 | 1.20 | 0.25 | 1.17 | 0.18 | 1.02 | 0.08 | 1.09 | 0.19 | 1.22 | 0.35 | 1.13 | 0.09 | ns |
| Asx | 1.79 | 0.15 | 1.77 | 0.13 | 1.45 | 0.32 | 1.45 | 0.65 | 0.73 | 0.37 | 1.20 | 0.29 | 0.78 | 0.32 | 1.43 | 0.44 | ns |
| Glx | 1.85 | 0.13 | 1.79 | 0.09 | 1.76 | 0.15 | 1.69 | 0.44 | 1.07 | 0.28 | 1.48 | 0.13 | 1.26 | 0.22 | 1.66 | 0.33 | ns |
| Hyp | 0.18 | 0.01 | 0.14 | 0.01 | 0.18 | 0.03 | 0.17 | 0.01 | 0.14 | 0.00 | 0.14 | 0.02 | 0.18 | 0.02 | 0.17 | 0.02 | ns |
| Phe | 0.36 | 0.01 | 0.34 | 0.02 | 0.36 | 0.02 | 0.29 | 0.05 | 0.23 | 0.06 | 0.32 | 0.03 | 0.30 | 0.04 | 0.27 | 0.04 | ns |
| Lys | 1.33 | 0.17 | 0.97 | 0.14 | 1.24 | 0.15 | 1.29 | 0.40 | 0.60 | 0.03 | 0.83 | 0.03 | 0.93 | 0.09 | 0.99 | 0.17 | ns |
| Tyr | 0.16 | 0.02 | 0.19 | 0.01 | 0.19 | 0.01 | 0.18 | 0.05 | 0.11 | 0.04 | 0.18 | 0.02 | 0.16 | 0.02 | 0.15 | 0.01 | ns |
| Total | 14.09 | 0.87 | 12.59 | 0.71 | 12.99 | 1.77 | 12.30 | 2.66 | 8.21 | 1.56 | 10.89 | 1.30 | 10.47 | 1.80 | 11.94 | 2.01 | ns |

Table S 6: δ^15^N values of individual amino acids from two-compartment N transfer experiments. Values are mean (*n* = 4).

|  | δ^15^N / ‰ | | | | | | | | | | | | | | | |
| --- | --- | --- | --- | --- | --- | --- | --- | --- | --- | --- | --- | --- | --- | --- | --- | --- |
|  | Control | | ^15^N-urea only | | Decomposition | | Exudation | | Defoliation | | Sterile | | Fungi | | Weevil | |
|  | Mean | SEM | Mean | SEM | Mean | SEM | Mean | SEM | Mean | SEM | Mean | SEM | Mean | SEM | Mean | SEM |
| Ala | 12.3 | 0.3 | 58.2 | 11.9 | 237.6 | 37.9 | 22.5 | 8.5 | 34.0 | 4.3 | 32.2 | 4.2 | 31.0 | 5.2 | 35.6 | 13.3 |
| Gly | 5.1 | 0.3 | 38.9 | 9.5 | 211.2 | 37.0 | 11.1 | 6.1 | 20.9 | 3.7 | 19.4 | 2.8 | 18.2 | 3.4 | 22.0 | 10.5 |
| Val | 10.0 | 0.3 | 53.8 | 11.4 | 224.1 | 42.9 | 16.5 | 8.4 | 31.9 | 6.9 | 23.8 | 3.6 | 23.7 | 5.2 | 21.8 | 4.8 |
| Leu | 7.5 | 0.8 | 71.5 | 15.3 | 330.9 | 68.9 | 20.2 | 10.6 | 39.3 | 12.4 | 30.2 | 5.2 | 35.0 | 7.8 | 35.2 | 13.4 |
| Thr | 4.5 | 0.5 | 46.3 | 11.5 | 216.8 | 26.1 | 15.7 | 6.5 | 28.1 | 3.6 | 21.5 | 3.3 | 24.1 | 2.8 | 31.3 | 18.2 |
| Ser | 4.0 | 0.2 | 51.0 | 13.4 | 249.7 | 40.4 | 12.6 | 7.7 | 25.6 | 5.7 | 21.2 | 3.7 | 23.0 | 4.1 | 31.4 | 15.2 |
| Pro | 9.5 | 0.2 | 59.9 | 13.0 | 266.1 | 36.2 | 18.3 | 7.8 | 34.6 | 5.0 | 31.5 | 4.8 | 35.5 | 5.6 | 46.6 | 16.6 |
| Asx | 10.0 | 0.2 | 56.2 | 13.1 | 245.0 | 36.2 | 18.9 | 7.2 | 32.6 | 2.2 | 23.7 | 3.7 | 28.5 | 3.7 | 37.5 | 13.6 |
| Glx | 11.3 | 0.4 | 79.1 | 17.6 | 348.2 | 37.9 | 26.5 | 12.8 | 41.3 | 3.5 | 32.9 | 5.1 | 37.2 | 6.5 | 45.7 | 18.2 |
| Hyp | 6.7 | 0.7 | 54.3 | 18.5 | 417.2 | 56.9 | 37.4 | 25.2 | 57.5 | 15.2 | 61.5 | 9.4 | 35.1 | 7.7 | 72.0 | 27.2 |
| Phe | 9.1 | 0.1 | 60.6 | 13.5 | 303.3 | 55.2 | 19.6 | 9.7 | 40.0 | 7.8 | 26.6 | 4.5 | 25.3 | 5.8 | 27.5 | 8.3 |
| Lys | 4.4 | 0.6 | 22.2 | 7.3 | 99.5 | 12.6 | 8.9 | 2.5 | 12.1 | 1.5 | 11.7 | 3.1 | 8.5 | 0.6 | 11.8 | 5.0 |
| Tyr | 5.8 | 0.1 | 60.5 | 15.9 | 241.9 | 40.1 | 14.8 | 7.8 | 32.9 | 7.8 | 26.5 | 5.0 | 24.8 | 6.0 | 30.5 | 14.0 |

Table S 7: Recovery rates of a standard amino sugar mixture added to sand and leached. Values are mean (n=4) and a dash (-) indicates there was no detectable loss.

| Amino Acid | Percentage Loss / % |
| --- | --- |
| Alanine | 3.22 |
| Glycine | - |
| Valine | - |
| Leucine | - |
| Threonine | - |
| Serine | - |
| Proline | - |
| Aspartate | 10.60 |
| Glutamate | 5.28 |
| Hydroxyproline | - |
| Phenylalanine | 5.23 |
| Lysine | 65.97 |
| Tyrosine | 24.30 |

Table S 8: Percentage total nitrogen and bulk δ^15^N values for reverse N transfer experiments. Values are mean (*n* = 4).

| Treatment | Pool | Control | | | | | ^15^N-urea (100 h) | | | | |
| --- | --- | --- | --- | --- | --- | --- | --- | --- | --- | --- | --- |
|  |  | % TN | SE | δ^15^N / ‰ | SE | % TN | | SE | δ^15^N / ‰ | SE |  |
| Clover to grass | Clover roots LC | 2.71 | 0.16 | 0.1 | 0.4 | 2.58 | | 0.23 | 49120 | 4178 |  |
|  | Clover shoots | 3.83 | 0.08 | 1.2 | 0.5 | 3.44 | | 0.08 | 8598 | 1409 |  |
|  | Clover roots TC | 2.64 | 0.26 | 1.7 | 0.6 | 2.65 | | 0.07 | 1719 | 316 |  |
|  | Soil TC | 0.51 | 0.004 | 6.5 | 0.2 | 0.49 | | 0.007 | 15.7 | 4.6 |  |
|  | Grass roots TC | 1.64 | 0.06 | 4.9 | 0.8 | 1.39 | | 0.07 | 24.7 | 4.5 |  |
|  | Grass shoots | 3.08 | 0.13 | 6.3 | 0.5 | 3.07 | | 0.38 | 6.5 | 0.5 |  |
|  | Grass roots RC | 1.67 | 0.13 | 5.2 | 0.6 | 1.55 | | 0.04 | 14.9 | 1.7 |  |
|  | Soil RC | 0.50 | 0.003 | 5.8 | 0.3 | 0.50 | | 0.008 | 5.1 | 1.0 |  |
| Grass to clover | Grass roots LC | 1.11 | 0.22 | 6.3 | 0.7 | 1.33 | | 0.09 | 53313 | 11353 |  |
|  | Grass shoots | 1.82 | 0.12 | 5.5 | 0.5 | 2.20 | | 0.17 | 19022 | 7280 |  |
|  | Grass roots TC | 1.38 | 0.17 | 6.0 | 1.2 | 1.16 | | 0.05 | 4136 | 1185 |  |
|  | Soil TC | 0.49 | 0.003 | 5.8 | 0.2 | 0.48 | | 0.004 | 22.3 | 7.9 |  |
|  | Clover roots TC | 2.64 | 0.09 | 5.4 | 1.4 | 2.59 | | 0.24 | 2149 | 786 |  |
|  | Clover shoots | 3.74 | 0.10 | 7.6 | 0.9 | 3.35 | | 0.15 | 10.6 | 0.9 |  |
|  | Clover roots RC | 2.69 | 0.17 | 5.9 | 0.5 | 2.89 | | 0.10 | 18.7 | 3.1 |  |
|  | Soil RC | 0.50 | 0.002 | 5.8 | 0.02 | 0.49 | | 0.008 | 6.6 | 0.1 |  |

Table S 9: Concentration of individual amino acids in transfer compartment (TC) and receiving compartment (RC) soil for transfer of ^15^N from clover to ryegrass. Values are mean (*n* = 4). P value indicates whether here was a significant difference between the control and ^15^N-urea treatments (t-test, P<0.05).

|  | TC | | | | | RC | | | | |
| --- | --- | --- | --- | --- | --- | --- | --- | --- | --- | --- |
|  | Control | | ^15^N-urea | | P | Control | | ^15^N-urea | | Sig |
| AA | Concentration / mg g^-1^ soil | SE / mg g^-1^ soil | Concentration / mg g^-1^ soil | SE / mg g^-1^ soil |  | Concentration / mg g^-1^ soil | SE / mg g^-1^ soil | Concentration / mg g^-1^ soil | SE / mg g^-1^ soil |  |
| Ala | 1.14 | 0.04 | 1.18 | 0.05 | NS | 1.38 | 0.11 | 1.50 | 0.11 | NS |
| Gly | 1.10 | 0.05 | 1.28 | 0.05 | NS | 1.34 | 0.07 | 1.57 | 0.13 | NS |
| Val | 0.54 | 0.05 | 0.56 | 0.04 | NS | 0.69 | 0.07 | 0.63 | 0.07 | NS |
| Leu | 0.80 | 0.04 | 0.69 | 0.01 | NS | 0.87 | 0.08 | 0.87 | 0.05 | NS |
| Thr | 0.98 | 0.10 | 1.15 | 0.11 | NS | 1.29 | 0.24 | 1.36 | 0.16 | NS |
| Ser | 0.64 | 0.04 | 0.75 | 0.04 | NS | 0.85 | 0.17 | 0.98 | 0.07 | NS |
| Pro | 0.87 | 0.03 | 1.10 | 0.04 | NS | 0.99 | 0.15 | 1.09 | 0.07 | NS |
| Asx | 1.35 | 0.10 | 1.47 | 0.11 | NS | 1.68 | 0.44 | 1.94 | 0.15 | NS |
| Glx | 1.47 | 0.07 | 1.56 | 0.05 | NS | 1.76 | 0.33 | 1.97 | 0.10 | NS |
| Hyp | 0.13 | 0.01 | 0.15 | 0.01 | NS | 0.16 | 0.01 | 0.18 | 0.02 | NS |
| Phe | 0.31 | 0.01 | 0.24 | 0.01 | NS | 0.32 | 0.04 | 0.28 | 0.01 | >0.05 |
| Lys | 0.53 | 0.02 | 1.43 | 0.15 | 0.003 | 0.75 | 0.19 | 1.21 | 0.22 | NS |
| Tyr | 0.13 | 0.01 | 0.15 | 0.01 | NS | 0.15 | 0.04 | 0.19 | 0.01 | >0.01 |
| Total | 9.99 | 0.56 | 11.69 | 0.69 | NS | 12.25 | 1.95 | 13.77 | 1.18 | NS |

Table S 10: Concentration of individual amino acids in transfer compartment (TC) and receiving compartment (RC) soil for transfer of ^15^N from ryegrass to clover. Values are mean (*n* = 4). P value indicates whether here was a significant difference between the control and ^15^N-urea treatments (t-test, P<0.05).

|  | TC | | | | | RC | | | | |
| --- | --- | --- | --- | --- | --- | --- | --- | --- | --- | --- |
|  | Control | | ^15^N-urea | | P | Control | | ^15^N-urea | | Sig |
| AA | Concentration / mg g^-1^ soil | SE / mg g^-1^ soil | Concentration / mg g^-1^ soil | SE / mg g^-1^ soil |  | Concentration / mg g^-1^ soil | SE / mg g^-1^ soil | Concentration / mg g^-1^ soil | SE / mg g^-1^ soil |  |
| Ala | 1.04 | 0.17 | 1.28 | 0.03 | NS | 1.21 | 0.03 | 1.53 | 0.16 | NS |
| Gly | 1.17 | 0.17 | 1.24 | 0.02 | NS | 1.27 | 0.06 | 1.52 | 0.21 | NS |
| Val | 0.40 | 0.14 | 0.49 | 0.02 | NS | 0.65 | 0.13 | 0.57 | 0.04 | NS |
| Leu | 0.90 | 0.10 | 0.76 | 0.01 | NS | 0.82 | 0.03 | 0.90 | 0.06 | NS |
| Thr | 0.74 | 0.32 | 1.00 | 0.04 | NS | 1.33 | 0.23 | 1.20 | 0.10 | NS |
| Ser | 0.50 | 0.20 | 0.72 | 0.01 | NS | 0.82 | 0.11 | 0.91 | 0.09 | NS |
| Pro | 1.22 | 0.21 | 0.95 | 0.01 | NS | 0.93 | 0.04 | 1.11 | 0.11 | NS |
| Asx | 0.79 | 0.45 | 1.59 | 0.06 | NS | 1.56 | 0.19 | 1.76 | 0.11 | NS |
| Glx | 1.06 | 0.37 | 1.74 | 0.04 | NS | 1.62 | 0.12 | 1.93 | 0.13 | NS |
| Hyp | 0.14 | 0.02 | 0.15 | 0.00 | NS | 0.15 | 0.01 | 0.17 | 0.02 | NS |
| Phe | 0.18 | 0.03 | 0.30 | 0.01 | 0.010 | 0.27 | 0.03 | 0.31 | 0.01 | NS |
| Lys | 0.66 | 0.08 | 0.98 | 0.16 | NS | 1.05 | 0.11 | 1.02 | 0.21 | NS |
| Tyr | 0.08 | 0.02 | 0.17 | 0.01 | 0.008 | 0.16 | 0.02 | 0.15 | 0.02 | NS |
| Total | 8.89 | 2.28 | 11.37 | 0.42 | NS | 11.83 | 1.10 | 13.07 | 1.27 | NS |

Table S 11: δ^15^N values of individual amino acids from the three-compartment N transfer experiment from clover to ryegrass in the transfer compartment (TC) and receiving compartment (RC). Values are mean (*n* = 4).

|  | TC | | | | RC | | | |
| --- | --- | --- | --- | --- | --- | --- | --- | --- |
|  | Control | | ^15^N-urea | | Control | | ^15^N-urea | |
| AA | δ^15^N value / ‰ | SE / ‰ | δ^15^N value / ‰ | SE / ‰ | δ^15^N value / ‰ | SE / ‰ | δ^15^N value / ‰ | SE / ‰ |
| Ala | 11.5 | 0.2 | 11.7 | 0.3 | 11.5 | 0.3 | 11.8 | 0.2 |
| Gly | 3.4 | 0.4 | 3.8 | 0.2 | 3.8 | 0.2 | 3.2 | 0.1 |
| Val | 10.7 | 1.6 | 10.3 | 0.6 | 12.2 | 0.3 | 9.0 | 0.6 |
| Leu | 7.2 | 0.2 | 6.9 | 0.1 | 7.1 | 0.2 | 6.3 | 0.5 |
| Thr | 4.3 | 0.4 | 3.9 | 0.4 | 5.1 | 0.1 | 2.8 | 0.1 |
| Ser | 3.2 | 0.2 | 4.0 | 0.3 | 3.6 | 0.1 | 3.8 | 0.2 |
| Pro | 8.7 | 0.4 | 21.5 | 0.1 | 9.2 | 0.8 | 8.8 | 0.3 |
| Asx | 9.2 | 0.5 | 12.0 | 0.2 | 9.2 | 0.3 | 9.6 | 0.2 |
| Glx | 9.5 | 0.3 | 13.6 | 0.3 | 10.1 | 0.8 | 10.5 | 0.3 |
| Hyp | 9.1 | 0.1 | 8.6 | 0.6 | 8.3 | 0.6 | 8.5 | 0.7 |
| Phe | 9.4 | 0.6 | 7.7 | 0.6 | 10.1 | 0.8 | 8.4 | 0.2 |
| Lys | 2.8 | 0.5 | 4.1 | 0.3 | 3.8 | 0.5 | 2.7 | 0.4 |
| Tyr | 3.9 | 0.4 | 5.8 | 0.3 | 4.6 | 0.2 | 5.2 | 0.3 |

Table S 12: δ^15^N values of individual amino acids from the three-compartment N transfer experiment from ryegrass to clover in the transfer compartment (TC) and receiving compartment (RC). Values are mean (*n* = 4).

|  | TC | | | | RC | | | |
| --- | --- | --- | --- | --- | --- | --- | --- | --- |
|  | Control | | ^15^N-urea | | Control | | ^15^N-urea | |
| AA | δ^15^N value / ‰ | SE / ‰ | δ^15^N value / ‰ | SE / ‰ | δ^15^N value / ‰ | SE / ‰ | δ^15^N value / ‰ | SE / ‰ |
| Ala | 11.2 | 0.2 | 27.8 | 7.3 | 10.9 | 0.1 | 12.1 | 0.2 |
| Gly | 3.6 | 0.1 | 13.0 | 4.8 | 3.8 | 0.2 | 4.1 | 0.2 |
| Val | 10.3 | 0.6 | 18.8 | 6.0 | 10.0 | 0.6 | 9.8 | 0.4 |
| Leu | 6.5 | 0.3 | 25.8 | 9.4 | 7.5 | 0.7 | 6.8 | 0.2 |
| Thr | 4.5 | 0.4 | 17.2 | 4.2 | 4.6 | 0.5 | 3.8 | 0.2 |
| Ser | 3.5 | 0.2 | 16.6 | 7.3 | 3.9 | 0.4 | 4.8 | 0.4 |
| Pro | 8.6 | 0.2 | 29.1 | 12.3 | 9.1 | 0.4 | 9.7 | 0.6 |
| Asx | 9.2 | 0.1 | 42.3 | 18.0 | 9.5 | 0.2 | 10.4 | 0.5 |
| Glx | 9.7 | 0.2 | 61.1 | 25.3 | 10.1 | 0.5 | 11.8 | 0.8 |
| Hyp | 8.8 | 0.9 | 17.4 | 4.9 | 7.6 | 0.5 | 8.9 | 0.2 |
| Phe | 8.6 | 0.4 | 25.1 | 8.5 | 8.7 | 0.5 | 8.2 | 0.8 |
| Lys | 3.4 | 0.5 | 5.4 | 1.2 | 3.6 | 0.6 | 3.8 | 0.4 |
| Tyr | 3.7 | 0.1 | 17.9 | 5.0 | 4.1 | 0.6 | 5.7 | 0.3 |
